# Pharmaceutical Manipulation of Mitochondrial F0F1‐ATP Synthase Enables Imaging and Protection of Myocardial Ischemia/Reperfusion Injury Through Stress‐induced Selective Enrichment

**DOI:** 10.1002/advs.202307880

**Published:** 2023-12-14

**Authors:** Zelin Chen, Xu Tan, Taotao Jin, Yu Wang, Linyong Dai, Gufang Shen, Can Zhang, Langfan Qu, Lei Long, Chongxing Shen, Xiaohui Cao, Jianwu Wang, Huijuan Li, Xiaofeng Yue, Chunmeng Shi

**Affiliations:** ^1^ Institute of Rocket Force Medicine State Key Laboratory of Trauma and Chemical Poisoning Army Medical University Chongqing 400038 China; ^2^ Department of Urology The Third Affiliated Hospital of Chongqing Medical University Chongqing 401120 China

**Keywords:** imaging, ischemia, mitochondria, protection, reperfusion

## Abstract

To rescue ischemic myocardium from progressing to myocardial infarction, timely identification of the infarct size and reperfusion is crucial. However, fast and accurate identification, as well as the targeted protection of injured cardiomyocytes following ischemia/reperfusion (I/R) injury, remain significantly challenging. Here, a near infrared heptamethine dye IR‐780 is shown that has the potential to quickly monitor the area at risk following I/R injury by selectively entering the cardiomyocytes of the at‐risk heart tissues. Preconditioning with IR‐780 or timely IR‐780 administration before reperfusion significantly protects the heart from ischemia and oxidative stress‐induced cell death, myocardial remodeling, and heart failure in both rat and pig models. Furthermore, IR‐780 can directly bind to F0F1‐ATP synthase of cardiomyocytes, rapidly decrease the mitochondrial membrane potential, and subsequently slow down the mitochondrial energy metabolism, which induces the mitochondria into a “quiescent state” and results in mitochondrial permeability transition pore inhibition by preventing mitochondrial calcium overload. Collectively, the findings show the feasibility of IR‐780‐based imaging and protection strategy for I/R injury in a preclinical context and indicate that moderate mitochondrial function depression is a mode of action that can be targeted in the development of cardioprotective reagents.

## Graphical abstract

This work identifies a near infrared heptamethine dye, IR‐780, exhibiting stress‐induced selective enrichment in the at‐risk heart tissues following ischemia/reperfusion (I/R) injury and further protecting heart from I/R and oxidative stress induced cell death by targeting the mitochondrial F0F1‐ATP synthase of the cardiomyocytes, which promises IR‐780 as a potential ischemia‐selective agent for imaging and protection of myocardial I/R injury.

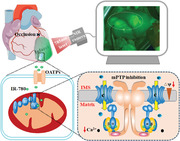

## 1 Introduction

Obstructive coronary artery disease‐induced myocardial infarction is one of the leading causes of morbidity and death worldwide.^[^ ^1^ ^]^ At present, the only method to rescue ischemic myocardium from myocardial infarction progression is the timely identification of the infarct size and reperfusion. However, in addition to salvage ischemic myocardium from infarction, reperfusion itself could also induce a specific additional component of irreversible injury.^[^ ^2^ ^]^ Thus, adjunct cardioprotection and reperfusion are urgently needed. Previously, several cardioprotective strategies including ischemic conditioning, ischemic conditioning‐derived pharmacological interventions, hypothermia, and vagal stimulation were developed but shown disappointingly poor translation into a clinical benefit in patients with acute myocardial infarction.^[^ ^3^ ^]^ The reason of the unsatisfactory cardioprotective strategies may be because of a poor understanding of the underlining pathologic mechanism and subsequently failing to accurately develop targeting drugs. Therefore, investigating the exact regulation signaling pathway following ischemia/reperfusion (I/R) and developing effective and preferably targeted cardioprotective strategy that can be administered before or during reperfusion remain major unmet medical needs.^[^ ^3b^ ^]^

The mitochondria, the key organelle for most of the cell's energy production, are also the center of cell death regulation.^[^ ^4^ ^]^ According to previous studies of ischemic conditioning, the mitochondria have been shown as one of the most significant ending targets of all cardioprotective signaling pathways.^[^ ^5^ ^]^ Thus, developing mitochondria‐targeting cardioprotective strategies holds great potential. Previous evidence has indicated that irreversible opening of the mitochondrial permeability transition pore (mPTP) is the key molecular event during I/R to induce necrosis,^[^ ^4^ ^]^ which has been shown to be the main mode of cell death following I/R.^[^ ^6^ ^]^ The mechanism studies of ischemic conditioning have shown that the protective effects of mitochondrial *K*_ATP_^[^ ^7^ ^]^ and connexin 43,^[^ ^8^ ^]^ which are key targets of ischemic conditioning, are related to mPTP inhibition by decreasing mitochondrial calcium (Ca^2+^) overload. Moreover, pharmaceutical mPTP opening inhibition before reperfusion could effectively decrease the infarcted area.^[^ ^9^ ^]^ Unfortunately, previous mPTP‐related cardioprotective drugs including cyclosporine A (CsA) have shown no significant cardioprotective effects in preclinical studies or clinical trials.^[^ ^10^ ^]^ The reason may be because of not fully understanding the molecular nature of the mPTP,^[^ ^11^ ^]^ thereby leading to the failure of a structure‐based drug design. Recent studies have shown that complex V of the mitochondrial oxidative phosphorylation (OXPHOS) electron transport chain (also named F0F1‐ATP synthase) is the key component of mPTP,^[^ ^12^ ^]^ suggesting that the mitochondrial OXPHOS electron transport chain is a potential cardioprotective target.^[^ ^13^ ^]^ It has shown cardioprotective effects by selectively binding with the S‐nitrosation of Cys39 on the ND3 subunit, thereby inhibiting complex I activation during reperfusion.^[^ ^14^ ^]^ Pharmaceutical inhibition of complex II activity during I/R has shown to be an effective cardioprotective strategy by succinate accumulation regulation during the ischemic stage and its oxidation during the subsequent reperfusion.^[^ ^15^ ^]^ However, whether complex V would be a potential cardioprotective target during I/R injury remains unclear owing to the lack of a direct complex V‐targeted agent or strategy.

Here, we characterized IR‐780, a near infrared (NIR) heptamethine dye, which could be quickly and selectively uptaken by the injured cardiomyocytes of the area at risk. NIR imaging revealed that IR‐780 would be a smart and practical probe for the early identification of infarcted areas. Furthermore, preconditioning with IR‐780 or timely IR‐780 administration before reperfusion significantly protects the heart from ischemia and oxidative stress‐induced cell death, myocardial remodeling, and heart failure in both rat and pig models. The protective effects of IR‐780 rely on binding to complex V of the mitochondrial OXPHOS electron transport chain, thereby rapidly decreasing the mitochondrial membrane potential and subsequently slowing down the mitochondrial energy metabolism, which induces the mitochondria into a “quiescent state.” Additionally, by inhibiting mitochondrial Ca^2+^ overload, IR‐780 partially inhibited mPTP opening. These results show that IR‐780 is a potential ischemia‐selective agent for the imaging and protection of myocardial I/R injury.

## 2 Results

### 2.1 IR‐780 Identifies the Area at Risk of Infarction

**Figure 1:**
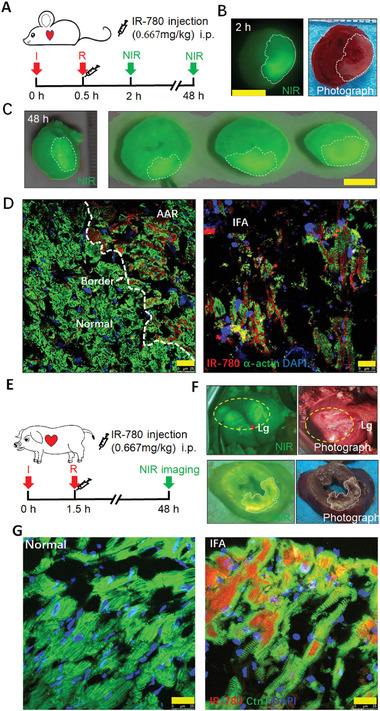
IR‐780 identifies the area at risk of infarction following I/R. A) Schematic of the IR‐780 based imaging protocols in rat I/R model. B) Left: representative image of the NIR imaging 2 h following reperfusion in rat I/R model. Right: the corresponding bright‐field photograph of the same tissue following TTC staining. Scale bar, 5 mm. C) Left: representative image of the NIR imaging of the whole heart 48 h following reperfusion in rat I/R model. Right: the corresponding NIR imaging of the transverse sections. Scale bar, 5 mm. D) representative confocal images of the injured heart tissues 48 h following reperfusion in rat I/R model. AAR: area at risk, IFA: infarcted area. Scale bar, 25 µm. E) Schematic of the IR‐780 based imaging protocols in pig I/R model. F) up: representative image of the in vivo NIR imaging and bright‐field photograph of the injured heart 48 h following reperfusion in pig I/R model; down: the corresponding NIR imaging and bright‐field photograph of the corresponding heart transverse sections. G) representative confocal images of the injured heart tissues 48 h following reperfusion in pig I/R model. Lg: ligation, AAR: area at risk, IFA: infarcted area, CtnT: Cardiac Troponin T. Scale bar, 25 µm.

IR‐780 is a heptamethine dye with NIR fluorescent properties.^[^ ^16^ ^]^ Thus, we first investigated the feasibility of its in vivo imaging. Following left coronary artery ligation of the pig heart, IR‐780 was intraperitoneally injected. Ten minutes later, the heart was harvested for NIR imaging. The ischemic area showed significantly low NIR signals (Figure S1A–C, Supporting Information), which indicated successful ligation and IR‐780 as a workable in vivo imaging dye. Next, we detected the dynamic changes of IR‐780 in the injured heart tissues. Interestingly, the NIR signals in the area at risk was significantly increased as injury aggravated in both rat ischemia and I/R models (as early as 2 h following reperfusion or 4 h following ischemia, when the infarct tissues could be detected using triphenyl tetrazolium chloride [TTC] staining) (**Figure** **1**A,B and Figure S2A,B, Supporting Information). The comparison rate of the area at risk by Evans blue and IR‐780 and the infarct size by TTC staining and IR‐780 are 96.79% ± 1.21 and 45.35% ± 4.34, respectively. Moreover, at 48 h following I/R, IR‐780 remained selectively retained in the infarcted area in both rat and pig I/R models (Figure 1C,E,F; Video S1, Supporting Information), when obvious morphological infarct injury emerged.^[^ ^17^ ^]^ Laser confocal images of the IR‐780‐labeled infarct tissues showed that IR‐780 signals were selectively accumulated in the cardiomyocytes of the area at risk and the infarcted area, whereas they were significantly low in the cardiomyocytes of the normal area (Figure 1D,G; Figure S2C, Supporting Information). Further, we tested the dynamic accumulation of IR‐780 in the injured heart tissues when IR‐780 was injected immediately following ischemia (Figure S3A, Supporting Information). Thirty minutes later, low IR‐780 signals were detected in the ischemia area (Figure S3B, Supporting Information), indicating IR‐780 infiltration even during ischemia. Following reperfusion, IR‐780 quickly accumulated in the injured heart tissues as early as 10 min (Figure S3C,D, Supporting Information). Moreover, the NIR signal could be detected even at 5 days following IR‐780 injection (Figure S4A,B, Supporting Information), which suggested the in vivo imaging stability of IR‐780. In addition, it showed much higher fluorescence intensity in the injured heart tissues than the main organs including liver, lung, spleen, kidney, muscle and intestine 2 h post the reperfusion, and the contrast value of fluorescence between the area at risk of heart and the main organs ranged from 1.07–6.15 (Figure S4C,D, Supporting Information). These results demonstrated that IR‐780 is a potential NIR fluorescent small molecule to quickly identify the myocardial area at risk by targeting the injured cardiomyocytes.

### 2.2 IR‐780 Binds to Plasma Albumin Proteins and Releases in the Acidic Microenvironment

**Figure 2:**
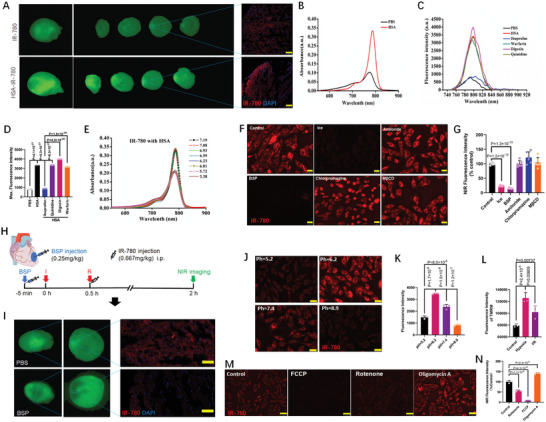
The uptake mechanism of IR‐780 by cardiomyocytes following I/R. A) Representative NIR imaging and confocal images of the heart tissues 2 h following reperfusion in rats treated with IR‐780 or HSA‐IR‐780. Scale bar: 75 µm. B) The absorbance of the IR‐780 dispersed in PBS and HSA. C,D) The albumin‐bond site of IR‐780 was determined with specific inhibitors (warfarin, ibuprofen, digoxin, and quinidine, used as competitive inhibitors of albumin‐binding sites I, II, III, and α1‐glycolipoprotein, respectively) (n = 3 per group). E) The absorbance changes of IR‐780 dispersed in HSA within different pH conditions. F,G) Representative images and quantitative data of the NIR fluorescent signals in H9C2 cells exposed to IR‐780 pre‐treatment with ice condition, sulfobromophthalein (BSP), amiloride (an actin inhibitor), chlorpromazine (a clathrin inhibitor) and MβCD (a caveolae inhibitor) (n = 3 per group). Scale bar: 75 µm. H) Schematic of the in vivo protocols to verify the roles of OATPs on the IR‐780 uptake. I) Representative NIR photographs of the whole heart (left panel), transverse sections (middle panel) and micro‐photographs of the heart tissue received IR‐780 injection with or without pre‐BSP local administration in rat I/R models. Scale bar: 75 µm. J,K) Representative images and quantitative flow cytometric data of the NIR fluorescent signals in H9C2 cells exposed to IR‐780 within different pH conditions (n = 3 per group). Scale bar: 50 µm. L) Quantitative flow cytometric data of the mitochondrial membrane potential of the H9C2 cells exposed to hypoxia and I/R (n = 3 per group). M,N) Representative images and quantitative flow cytometric data of the NIR fluorescent signals in H9C2 cells exposed to IR‐780 pre‐treatment with FCCP, Rotenone, Oligomycin A (n = 3 per group). Scale bar: 50 µm. All the *p* values are present in the graphs.

We subsequently investigated how IR‐780 accumulates in the injured heart tissues. A previous study has reported that some heptamethine indocyanine dyes exhibit high affinity to albumin proteins in the plasma, thereby resulting in enhanced fluorescence and permeability, and retention effects.^[^ ^18^ ^]^ First, we developed human serum albumin proteins‐IR‐780 (HSA‐IR‐780) nanoparticles, and the injection of these nanoparticles following reperfusion enhanced the NIR signals in the injured heart tissues following I/R injury (**Figure** **2**A). Second, the absorbance spectra of IR‐780 dissolved in phosphate‐buffered saline (PBS) with HSA were significantly increased (Figure 2B). Moreover, the albumin‐bond site of IR‐780 was further determined using specific inhibitors (warfarin, ibuprofen, digoxin, and quinidine). Of them, ibuprofen (an albumin binding site II inhibitor) significantly decreased the ‐fluorescence intensity of the IR‐780 solutions with HSA (Figure 2C,D). We further incubated the H9C2 cells with IR‐780 solutions with or without HSA or ibuprofen; we observed a decreased uptake of IR‐780 with HSA, which was recovered by ibuprofen (Figure S5A,B, Supporting Information). During ischemia, a metabolic switch to increase lactate production causes a decrease in tissue pH.^[^ ^19^ ^]^ With the decrease in pH levels from 7.19 to 3.38, the characteristic spectra of HSA binding IR‐780 complexes shifted to that of free IR‐780 beginning at low pH levels (≈6.23) (Figure 2E), suggesting that heart ischemic acidic microenvironment benefits from its specific release. The abovementioned data suggest that IR‐780 binds to plasma albumin proteins and is released into the acidic microenvironment associated with ischemia.

### 2.3 Uptake of IR‐780 by Cardiomyocytes is Dependent on Organic Anion Transporting Polypeptides (OATPs) and Mitochondrial Membrane Potential

Next, we investigated the uptake of IR‐780 by cardiomyocytes in the at‐risk heart tissues. In vitro, a significantly decreased IR‐780 uptake was observed with ice incubation, indicating that the cellular uptake of IR‐780 is an energy‐dependent process (Figure 2F,G). Furthermore, sulfobromophthalein (BSP) (an OATP inhibitor) remarkably affected the cellular uptake of IR‐780, suggesting that IR‐780 uptake was actively mediated by OATP transporters. However, pretreatments with various endocytotic inhibitors including amiloride (actin inhibitor), chlorpromazine (clathrin inhibitor), and methyl‐β‐cyclodextrin (MβCD, caveolae inhibitor) had no effects on IR‐780 uptake (Figure 2F,G), suggesting that IR‐780 uptake was not mediated by endocytotic mechanism. Furthermore, the local injection of BSP into the left ventricular front wall 5 min before rat I/R significantly decreased IR‐780 signals detected by heart tissue NIR imaging and laser confocal imaging (Figure 2H,I). These data indicate that OATPs mainly mediated IR‐780 uptake by cardiomyocytes. A previous study has reported that OATP activity is closely related to pH levels.^[^ ^20^ ^]^ We subsequently detected IR‐780 uptake under four pH conditions from 5.2 to 8.9. IR‐780 uptake was significantly increased in a pH level of 6.2, whereas it was decreased in pH levels of 5.2 and 8.9 (Figure 2J,K), indicating that the OATP activity was increased in a weakly acidic microenvironment associated with ischemia. Further, we verified that IR‐780 uptake was significantly increased when exposed to hypoxic conditions, whereas it was decreased when exposed to hypoxia and high pH levels (8.9) (Figure S5C,D, Supporting Information). Moreover, during ischemia, the mitochondrial function was inhibited and exhibited high mitochondrial membrane potential (Figure 2L). The H9C2 cells treated with carbonyl cyanide 4‐(trifluoromethoxy) phenylhydrazone (FCCP, an uncoupler of OXPHOS) and rotenone (a complex I inhibitor) exhibited significantly decreased IR‐780 uptake, whereas it was increased when treated with oligomycin A (a mitochondrial F0/F1‐ATPase inhibitor) (Figure 2M,N). These data indicate that IR‐780 uptake by cardiomyocytes is also dependent on the mitochondrial membrane potential.

### 2.4 IR‐780 Protects the Heart from I/R‐Induced Myocardial Death and Heart Failure

**Figure 3:**
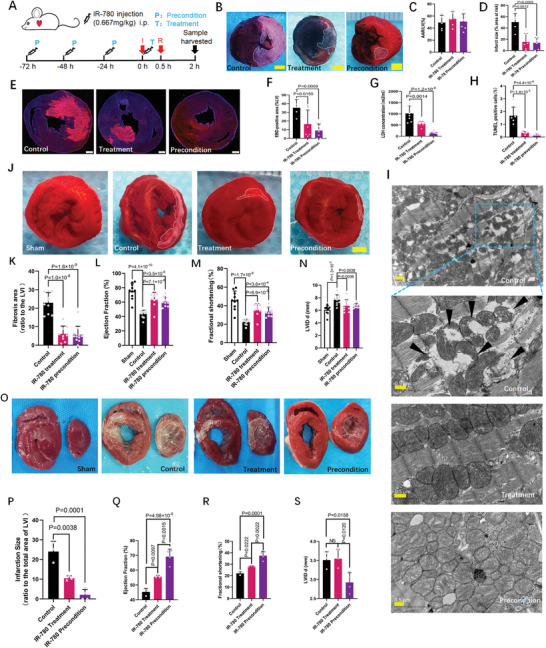
IR‐780 protects the heart from the I/R induced cell death and heart failure. A) Schematic of the IR‐780 based cardio‐protective protocols. B–D) Representative photographs and quantitative data for infarct size, area at risk (AAR) in rats of control (n = 5), IR‐780 treatment (n = 6) and IR‐780 precondition (n = 7) groups subjected to I/R; scale bar, 2 mm. E, F) Representative photomicrographs and quantitative data from images of myocardial EBD uptake in rats of control (n = 5), IR‐780 treatment(n = 6) and IR‐780 precondition (n = 8) groups subjected to I/R injury. Scale bar, 1 mm. G) Serum LDH concentrations in rats of control (n = 8), IR‐780 treatment (n = 7) and IR‐780 precondition (n = 7) groups with I/R injury. H) Quantitative data of TUNEL staining images in rats of control, IR‐780 treatment and IR‐780 precondition groups with I/R injury (n = 6 per group). I) Representative Transmission Electron Microscope (TEM) photomicrographs of the heart tissues in rats of control, IR‐780 treatment and IR‐780 precondition groups with I/R injury. Scale bar, 0.5 µm; arrows, rupture mitochondria. J,K) Representative photographs and quantitative data of the fibrosis area in rats of control (n = 9), IR‐780 treatment (n = 9) and IR‐780 precondition (n = 12) groups with 30 min cardiac ischemia and 4 weeks reperfusion. Scale bar, 2 mm. L–N) Averaged data ejection fraction (EF, L), fractional shortening (FS, M) and systolic left ventricle internal diameter (LVID d, N) assessed by echocardiography in rats of sham(n = 10), control (n = 10), IR‐780 treatment (n = 9) and IR‐780 precondition (n = 10) groups with sham or 30 min cardiac ischemia and 4 weeks reperfusion. O,P) Representative photographs and quantitative data of the fibrosis area in male pigs (4‐month‐old weighing 28–32 kg) of control (n = 3), IR‐780 treatment (n = 3) and IR‐780 precondition (n = 4) groups with 90 min cardiac ischemia and 4 weeks reperfusion. Scale bar, 2 mm. Q–S), Averaged data ejection fraction (EF, Q), fractional shortening (FS, R) and systolic left ventricle internal diameter (LVID, S) assessed by echocardiography in rats of sham(n = 2), control (n = 3), IR‐780 treatment (n = 3) and IR‐780 precondition (n = 4) groups with sham or 90 min cardiac ischemia and 4 weeks reperfusion. All the *p* values are present in the graphs.

IR‐780 has been shown to target ischemic cardiomyocytes; subsequently, we further asked whether IR‐780 could affect the cell fate of these cardiomyocytes following I/R. Surprisingly, both IR‐780 administered 72 h before ischemia and immediately following ischemia (**Figure** **3**A) markedly decreased the I/R‐induced myocardial infarct size from 50.4% to 13.7% and 15.4% of the area at risk, respectively; however, the groups had comparable areas at risk (Figure 3B–D). Notably, IR‐780‐preconditioned and treatment hearts were resistant to I/R‐induced myocardial necrosis, as evidenced by the sharp decrease in Evans blue dye (EBD) penetration and lactate dehydrogenase (LDH) release (Figure 3E–G). Additionally, the results of TUNEL staining showed the protective effects of IR‐780 preconditioning and treatment on I/R‐induced myocardial apoptosis (Figure 3H;Figure S6A, Supporting Information). Consistent with those of previous studies,^[^ ^6^ ^]^ our results also indicated a relatively lower proportion of apoptosis than that of necrosis following I/R. Moreover, transmission electron microscope images showed that IR‐780 preconditioning and treatment significantly reduced mitochondrial rupture and myocardial fiber breakage following I/R (Figure 3I), whereas, in rats without I/R, no significant morphologic changes were observed following IR‐780 preconditioning or treatment (Figure S6B, Supporting Information). In an ischemic injury rat model (4 h ischemia without reperfusion), IR‐780 preconditioning and treatment also significantly decreased the ischemia‐induced myocardial infarct size from 45.15% to 6.7% and 15.4%, respectively (Figure S7A–C, Supporting Information). Moreover, the decreased mitochondrial rupture or swelling following ischemia was shown in IR‐780‐preconditioned and treatment hearts (Figure S7D, Supporting Information). These results suggest that besides imaging, IR‐780 is also an effective cardioprotective agent for decreasing the I/R‐induced myocardial cell death.

As cardiomyocyte cell death plays significant roles in the pathological process of adverse cardiac remodeling and heart failure.^[^ ^4^, ^21^ ^]^ we subsequently evaluated the potential effects of IR‐780 on these sequelae of I/R injury. In a rat chronic I/R experimental setting (30 min ischemia followed by 4‐week reperfusion), IR‐780 preconditioning and treatment effectively blocked I/R‐induced cardiac fibrosis (Figure 3J,K). Furthermore, the I/R‐induced cardiac contractile dysfunction (as indicated by decreases in fractional shortening and ejection fraction) and left ventricular dilation (as assessed by systolic left ventricular internal diameter) were significantly abolished by IR‐780 preconditioning and treatment (Figure 3L–N;Figure S8A, Supporting Information). Notably, we evaluated the protective effects of IR‐780 in a pig chronic I/R model (90‐min ischemia followed by 4‐week reperfusion). Interestingly, IR‐780 preconditioning nearly completely abolished the I/R‐induced cardiac fibrosis area from 24.1% to 2.1%, and two of the four pigs in the IR‐780 precondition group showed no fibrosis (Figure 2O,P). Furthermore, the I/R‐induced cardiac fibrosis area was significantly decreased by IR‐780 treatment from 24.1% to 10.1% (Figure 2O,P). Moreover, IR‐780 preconditioning completely restored the cardiac contractile dysfunction and left ventricular dilation; IR‐780 treatment improved the cardiac contractile dysfunction (Figure 2Q–S;Figure S8B, Supporting Information). In addition, results of the blood routine, liver function, renal function indicated no significant changes of the main organ functions 1 month following IR‐780 administration in both pig and rat I/R models (Figures S9 and S10, Supporting Information). Therefore, the in vivo data of both acute and chronic models of I/R or ischemia injury indicate that IR‐780 is a potential candidate for protecting I/R or ischemia‐induced myocardial cell death and the sequelae of adverse cardiac remodeling and heart failure.

### 2.5 IR‐780 Targets the Mitochondria and Depresses the Mitochondrial Membrane Potential

**Figure 4:**
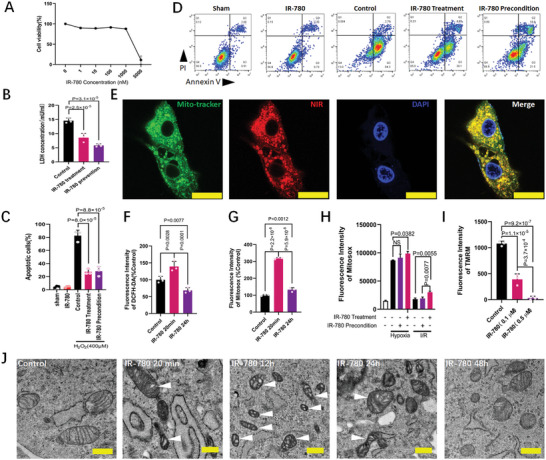
IR‐780 targets the mitochondria of the cardiomyocytes and affects the mitochondrial function. A) Cell viability assays for H9C2 cells exposed to increasing doses of IR‐780 over the course of 24 h (n = 6 pre group). B) LDH concentrations of cell culture medium in cells of control, IR‐780 treatment and IR‐780 precondition groups with 2 h ischemia and 1.5 h reperfusion (n = 6 per group). C,D) Representative images and quantitative data of the apoptosis in cells of sham, IR‐780, control, IR‐780 treatment and IR‐780 precondition groups with or without 400 µm H_2_O_2_ for 2 h (n = 3 per group). E) Representative confocal images of the H9C2 cells treated with IR‐780 (0.1 µm) and co‐staining with Mito‐tracker green. Scale bar, 25 µm. F,G) Total cell (F) and mitochondrial (G) ROS production of H9C2 cells 20 min and 24 h exposed to IR‐780 (0.1 µm), (n = 3 per time point). H) mitochondrial ROS production of H9C2 cells in control, IR‐780 treatment, IR‐780 precondition groups exposed to sham or ischemia (2 h) or ischemia (2 h) and reperfusion (5 min), n = 3 per group. I) Mitochondrial membrane potential test (TMRM staining) of H9C2 cells exposed to IR‐780 (0.1 or 0.5 µm) for 20 min, n = 3 per group. J) Representative TEM photomicrographs of H9C2 cells exposed to IR‐780 (0.1 µm) over the course of 48 h. Scale bar, 0.5 µm. All the *p* values are present in the graphs.

Subsequently, we investigated how the protective effects of IR‐780 (Figure S11A, Supporting Information) work in cardiomyocytes. We first evaluated the IC50 of IR‐780, which was ≈2.5 µm (**Figure** **4**A). Thus, we selected a relatively low concentration of 0.1 µm for the following in vitro experiments. Additionally, IR‐780 preconditioning and treatment protects the H9C2 cells from I/R‐induced necrosis and H_2_O_2_‐induced apoptosis, as evidenced by the significant reduction of LDH release and PI/Annexin V positive proportion, respectively (Figure 4B–D). As IR‐780 is an NIR fluorescent small molecule, we further investigated the subcellular localization of IR‐780 by detecting its fluorescent signals. Results indicated that IR‐780 superiorly accumulated in the mitochondria as shown by co‐staining with MitoTracker green (Figure 4E;Figure S11B, Supporting Information). A previous study has indicated that the mitochondria are one of the major sources of reactive oxygen species (ROS), which is one of the significant factors for inducing cell death during I/R.^[^ ^22^ ^]^ We evaluated whether IR‐780 could inhibit ROS production. Reversely, IR‐780 increased the mitochondrial and total cellular ROS production (Figure 4F,G;Figure S11C,E, Supporting Information). Twenty‐four hours following IR‐780 administration, the total cellular ROS significantly decreased, whereas the mitochondrial ROS remained higher than the control (Figure 4F,G). Data of the in vitro hypoxia or I/R models have also indicated no reduction of mitochondrial ROS in both IR‐780‐preconditioned and treatment groups (Figure 4H). Notably, IR‐780 administration sharply decreased the mitochondrial membrane potential even at a very low concentration of 0.1 µm (Figure 4I;Figure S11D,F, Supporting Information). The mitochondrial membrane potential could recover to the normal level at ≈6 h following a low‐dose IR‐780 administration (Figure S11D, Supporting Information). A previous study reported that mitochondrial membrane potential reduction could induce the subsequent mitochondrial ROS burst.^[^ ^22^ ^]^ Conversely, excess mitochondrial ROS accumulation also decreased the mitochondrial membrane potential.^[^ ^23^ ^]^ Our results of the mitochondrial ROS and mitochondrial membrane potential showed that the mitochondrial membrane potential decrease was accompanied with mitochondrial ROS increase, whereas the mitochondrial membrane potential recovered more rapidly than the mitochondrial ROS (Figure S11C,D, Supporting Information), suggesting that the mitochondrial membrane potential reduction causes the mitochondrial ROS increase. A previous study has shown that the decrease in the mitochondrial membrane potential could induce mitochondrial swelling.^[^ ^24^ ^]^ IR‐780 induced mitochondrial swelling at 20 min and was sustained until ≈24 h (Figure 4J). These results suggest that the protective effects of IR‐780 are mediated by mitochondrial membrane potential regulation.

### 2.6 IR‐780 Disrupts the Cell Metabolism of the Cardiomyocytes

**Figure 5:**
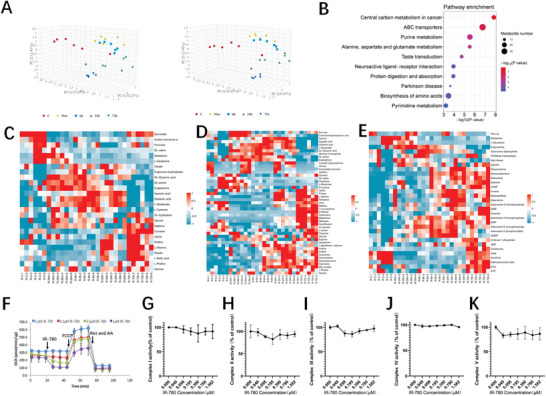
IR‐780 disrupts cell metabolism of the cardiomyocytes. A) The partial least squares‐discriminant analysis (PLS‐DA) score plot showing group distribution based on metabolite profiles of cell culture media samples of H9C2 cells exposed to IR‐780 (0.1 µm) over the course of 72 h, n = 6 per time point. B) Kyoto Encyclopedia of Genes and Genomes (KEGG) pathway enrichment analysis identified metabolites with −log10 (p value). The top 10 enriched pathways are shown. C–E) Heatmap of identified metabolites in the central carbon metabolism in cancer (C), ABC transporters (D) and Purine metabolism (E) pathways, n = 6 samples per group. F) Seahorse analyzer assays show that H9C2 cells undergo acute OCR inhibition. IR‐780 (introduced after 18 min) at four doses (0, 0.1, 0.5 and 1 µm; represented by four colored lines); FCCP (introduced after 48 min); and a mixture of rotenone and antimycin A (Rot and AA; introduced after 72 min). G‐K) Mitochondrial complex activities assay for isolated mitochondria exposure to different IR‐780 concentrations, n = 3 per concentration. All the *p* values are present in the graphs.

As the mitochondrial membrane potential is the basement of cell energy metabolism, we next performed a non‐targeted metabolomics analyses on cardiomyocytes at 20 min, 6, 24, and 72 h following IR‐780 administration. The results of the metabolic profiles were clearly segregated in the different groups based on PLS‐DA analysis (**Figure** **5**A). Pathway analysis identified central carbon metabolism in cancer, ABC transporters, and purine metabolism as the three greatest enrichment among the altered metabolites (Figure 5B). Importantly, among the identified metabolites involved in the three pathways, metabolites involved in tricarboxylic acid cycle and subsequent OXPHOS including pyruvate, acetyl coenzyme A, succinate, ATP, and ADP were significantly decreased following IR‐780 administration, whereas the metabolites in glutamine, uridine, and purine metabolisms were markedly increased (Figure 5C–E). Further analysis of the OXPHOS, glycolysis, and tricarboxylic acid cycle pathways indicated significant inhibition following IR‐780 administration (Figure S11H–J, Supporting Information). Notably, these changes recovered ≈24 h following IR‐780 administration (Figure 5C–E; Figure S11H–J, Supporting Information). Subsequently, we performed oxygen consumption rate (OCR) measurements and noted a dose‐dependent IR‐780 inhibition that was equally as potent as that of well‐characterized OXPHOS inhibitors (Figure 5F). However, prolonged (72 h) OCR measurements showed that cardiomyocytes fully recovered from this depletion (Figure S11G, Supporting Information). These data indicate that low‐dose IR‐780 induced the mitochondria into a “quiescent state” and reprogramed the glucose metabolism into glutamine, uridine, and purine metabolisms, suggesting that the compensative metabolism pathways play positive roles in the protection of cardiomyocytes from I/R injury by potentially fueling the stressed cardiomyocytes, as recently reported.^[^ ^25^ ^]^

### 2.7 IR‐780 Interacts with Respiratory Chain Complex V Proteins

**Figure 6:**
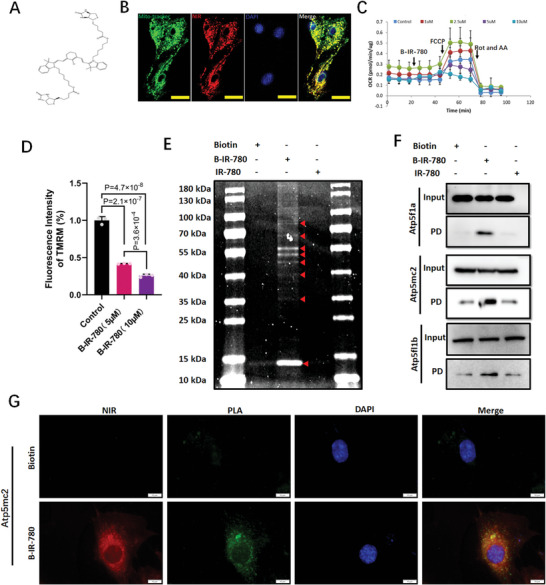
B‐IR‐780 interacts with complex V proteins of OXPHOS. A) B‐IR‐780 structure. B) Representative confocal images of the H9C2 cells treated with B‐IR‐780 (1 µm) and co‐staining with Mito‐tracker green. Scale bar, 25 µm. C) B‐IR‐780 induced OCR inhibition. B‐IR‐780 (0, 0.1, 5, 10 µm) was added after 18 min, followed by FCCP (added after 48 min) and a mixture of rotenone and antimycin A (72 min). n = 3. D) Mitochondrial membrane potential test (TMRM staining) of H9C2 cells exposed to IR‐780 (5 or 10 µm) for 20 min. n = 3 per group. E) Sodium dodecyl sulfate gel protein Coomassie Blue stain from B‐IR‐780 pull‐down assay using H9C2 cells treated with Biotin, B‐IR‐780, IR‐780, which demonstrates IR‐780‐specific interactions. N = 3. F) Biotin pull‐down followed by western blot with specific antibodies for atp5f1a, atp5f1b, and atp5mc2 validates complex V protein interaction with the IR‐780 moiety of B‐IR‐780. G) Proximity Ligation Assay for H9C2 cells exposed to biotin or B‐IR‐780 demonstrates the IR‐780‐specific interactions with complex V subunit atp5mc2. All the *p* values are present in the graphs.

As OXPHOS is accomplished by respiratory chain complexes *I*–*V*, we further estimated the effects of IR‐780 on the activities of respiratory chain complexes. Complex V, also called mitochondrial F0F1 ATP synthase, was mostly inhibited complex even at a very low concentration of 0.05 µm (≈20% decrease) (Figure 5G–K). We synthesized an active analog that was amenable to biotin moiety modification, which we named B‐IR‐780 (IC50 approximately equal to 4.5 µm for H9C2 cells), to further investigate the targets of IR‐780 (**Figure** **6A**;Figure S12A, Supporting Information). B‐IR‐780 conserves IR‐780 properties, including mitochondrial targeting, OCR inhibition, ROS induction, and reduction in mitochondrial membrane potential (Figure 6B–D; Figure S12B,C, Supporting Information). Biotin‐mediated pull‐down experiments using B‐IR‐780‐pretreated H9C2 cells, followed by electrophoresis, resolved multiple Coomassie blue‐stained bands that were absent in biotin‐ or IR‐780‐incubated H9C2 cells, indicating IR‐780‐specific interactions (Figure 6E). We conducted mass spectrometry analysis of eluate from pull‐down samples from the mitochondria of B‐IR‐780‐treated H9C2 cells and detected 33 mitochondrial proteins, of which eight were components of OXPHOS machinery ; components covering complexes III, IV, and V, and complex V was best represented (six proteins) (Figure S12D, Supporting Information). Of them, the ATP synthesis subunits α, β, and the proton channel subunit, atp5mc2, were listed. Next, we used the whole cell lysis pull‐down assay to verify the interactions between the IR‐780 and complex V components. Results showed significant interactions between B‐IR‐780 and ATP synthesis subunits α, β, and atp5mc2 (Figure 6F), whereas it failed to pull down the other three components (data not shown). Moreover, the results of the PLA experiment supported the interactions between B‐IR‐780 with these three components of complex V (Figure 6G;Figure S12E, Supporting Information). The abovementioned data indicate that complex V is the key target of IR‐780.

### 2.8 IR‐780 Inhibits the mPTP via Decreasing the Mitochondrial Calcium

**Figure 7:**
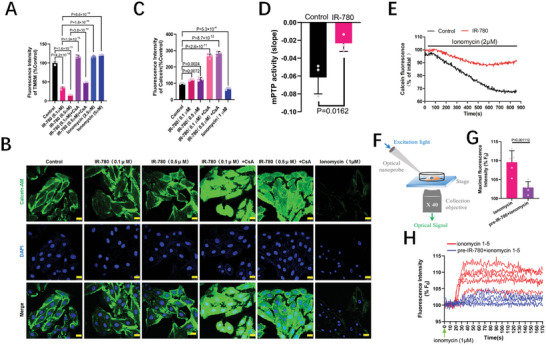
IR‐780 inhibits the mPTP activity and decreases the mitochondrial calcium. A) Mitochondrial membrane potential test (TMRM staining) of H9C2 cells exposed to sham, IR‐780 (0.1 or 0.5 µm) with or without pre‐treatment of cyclosporin A (CsA, 10 µm), ionomycin (1or 2.5 µm). B,C) Representative confocal images (B) and quantitative flow cytometry data (C) of the mitochondrial Calcein in the H9C2 cells exposed to sham, IR‐780 (0.1 or 0.5 µm) with or without pre‐treatment of cyclosporin A (CsA, 10 µm), ionomycin (1 µm). Scale bar, 25 µm, n = 3 per group. D,E) IR‐780 inhibits the mPTP activity. The mPTP activity assay (the decrease slope of the mitochondrial Calcein) (D) and representative traces of mitochondrial Calcein (E) during time monitored by the fluorescence of H9C2 cells exposed to ionomycin(1 µm) with or without pre‐IR‐780 (0.1 µm) (15 min before ionomycin administration), n = 3 per group. F) Schematic of the single cell‐based detecting of the fluorescence of 4mt‐GCamp6 in H9C2 cells. G,H) The maximal fluorescence of 4mt‐GCamp6 of each single cell (G) and the traces of mitochondrial Ca^2^+ concentration during time monitored by the fluorescence of 4mt‐GCamp6 of each single cell (H) exposed to ionomycin(1 µm) with or without pre‐IR‐780 (0.1 µm) (15 min before ionomycin administration), n = 5 per group. All the *p* values are present in the graphs.

Next, we identified the missing link between the protective effects of IR‐780 and its interactions with complex V, which is well known to phosphorylate ADP and generate ATP by exploiting the proton gradient generated by complexes I, III, and IV.^[^ ^26^ ^]^ Furthermore, complex V is considered to be the one of the key components of the mPTP.^[^ ^3^, ^12^, ^27^ ^]^ The irreversible opening of the mPTP has been proven to be the leading cause of cardiomyocyte necrosis during I/R.^[^ ^4^ ^]^ We subsequently investigated whether IR‐780 protects cardiomyocytes from I/R injury via influencing the mPTP function. CsA, a well‐accepted mPTP inhibitor, significantly reversed the IR‐780‐induced mitochondrial membrane potential decrease (**Figure** **7**A and Figure S13A, Supporting Information). Interestingly, in another mPTP opening assessment using the calcein–cobalt quenching assay,^[^ ^28^ ^]^ IR‐780 increased the calcein fluorescence intensity, whereas ionomycin, a Ca^2+^ ionophore and effective activator of mPTP, decreased it (Figure 7B,C;Figure S14E,F, Supporting Information). The results in freshly isolated mitochondria from adult rat heart is consistent with that in cells (Figure S14A,B, Supporting Information). However, Mito tracker, also a mitochondrial accumulated dye, has no effect on the mPTP activity (Figure S14C,D, Supporting Information). Surprisingly, ionomycin did not decrease the mitochondrial membrane potential (Figure 7A;Figure S13B, Supporting Information), and CsA could both inhibit mPTP opening and mitochondrial membrane potential decrease. Further, we continuously monitored the calcein fluorescence intensity of the cells with or without IR‐780 pretreatment followed by ionomycin administration. Data showed that the slope of the calcein fluorescence intensity reduction was significantly inhibited in the IR‐780‐pretreated cells (Figure 7D,E). These data indicate that the mPTP and the proton channel (known as ATP synthase c‐Subunit) targeted by IR‐780 are two independent channels, and both are regulated by CypD. IR‐780‐mediated proton channel opening could inhibit the mPTP opening. A previous study has reported that high Ca^2+^ concentrations in the mitochondrial matrix is the major stimulus for mPTP opening during I/R.^[^ ^4^ ^]^ Thus, we evaluated whether IR‐780 could influence Ca^2+^ concentrations in the mitochondrial matrix. Results of the adult rat cardiomyocytes staining with Rhod‐2 showed that IR‐780 could reduce the fluorescence intensity of Rhod‐2 in cells with and without ionomycin (Figure S14G,H, Supporting Information). We subsequently generated an H9C2 cell line expressing a mitochondrial localized, genetically encoded Ca^2+^ indicator, 4mt‐GCaMP6, whose fluorescence intensity reflects the free Ca^2+^ level in the mitochondria. Moreover, we developed a single‐cell optical signal detecting system, which could detect the real‐time optical signal dynamic at a single‐cell level (Figure 7F). Using this system, we monitored the real‐time mitochondrial Ca^2+^ dynamics of H9C2 cells with or without IR‐780 pretreatment following ionomycin administration. Results showed that H9C2 cells with IR‐780 pretreatment significantly decreased the maximal mitochondrial Ca^2+^ concentrations and delayed the time to reach the top following the ionomycin treatment (Figure 7G,H). Furthermore, using a traditional spinning disk confocal super resolution microscope in a real‐time model, we verified the results. Consistent with the results detected using the single‐cell system, data from the microscope also showed that IR‐780 significantly decreased the mitochondrial Ca^2+^ concentrations and inhibited the mitochondrial Ca^2+^ overload (Figure S15A–D, Supporting Information). These results indicate that IR‐780 could inhibit mPTP opening by preventing mitochondrial Ca^2+^ overload.

## 3 Conclusion

The infarct size is a major determinant of the outcomes and prognosis of patients with acute myocardial infarction.^[^ ^29^ ^]^ It depends on the size of the ischemic area at risk of infarction. Thus, accurate identification of the area at risk and infarct size as early as possible is of significant benefit for the following therapy. In this study, we developed a novel methodology for assessing the area at risk of infarction at the early stage of the condition (as early as 10 min). The current methods for evaluating the area at risk following I/R injury, which mainly include positron emission tomography /computed tomography with radionuclides such as sodium [18F] fluoride,^[^ ^30^ ^]^ radionuclides labeled molecules with injured related phospholipids binding activities,^[^ ^31^ ^]^ and T2‐weighted cardiac magnetic resonance imaging (T2‐CMR).^[^ ^32^ ^]^ Major limitations of the first two methodologies include adverse effects to hazardous ionizing radiation, intrinsically limited spatial resolutions, reconstruction‐dependent poor temporal resolution, high cost and lack of both exogenous and endogenous probes for molecular or functional imaging.^[^ ^33^ ^]^ The T2‐CMR is based on the edema at the injured sites. However, the experimental evidence points to substantial edema occurring in the infarcted region, with minimal or no edema occurring in the portion of the area at risk for a reversible injury.^[^ ^34^ ^]^ NIR fluorescence imaging within the wavelength range of 700–900 nm (the “biological transparency NIR window”) has attracted immense attention for in vivo imaging in both fundamental scientific research and clinical practice. In contrast with the aforementioned tomographic imaging modalities, NIR fluorescence imaging does not suffer from the same drawbacks, and instead provides the benefits of real‐time wide‐field image acquisition and diffraction‐limited, high sensitivity, high spatial resolution in living organisms. ^[^ ^16^, ^33^, ^35^ ^]^ However, there still lacks of favorable NIR probe for real time monitoring myocardial ischemia. In this study, we identified that IR‐780 as a stess‐indcued selective enrichment agent in the area at risk of myocardial infarction, which suggesting IR‐780 could be served as a myocardial injury detective probe. This is the first account of an ischemia‐selective cardioprotective fluorescent agent, utilizing the pathological differences between risk and non‐risk cardiomyocytes to drive uptake. However, to develop advanced NIR imaging devices, more effort is required.

**Figure 8:**
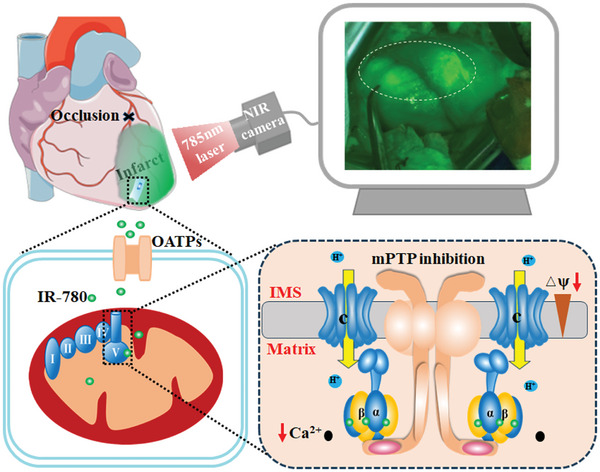
Schematic illustration of IR‐780 based imaging and cardioprotection of myocardial I/R injury. IR‐780 based imaging: IR‐780 is administrated immediately after reperfusion, and IR‐780 directly binds to plasma albumin proteins, go into the area at risk through  enhanced permeability and retention effects and releases in the acidic microenvironment associated with ischemia. Then, accumulated in the ischemic cardiomyocytes of the infarct area as the injury aggravation, and the infarct size could be assessed by a mobile NIR image Scope system. IR‐780 based cardioprotection: IR‐780 is administrated 72 h before ischemia or immediately after ischemia. Then, IR‐780 is increased uptake by the ischemic cardiomyocytes through enhanced activity of OATPs within the acidic microenvironment associated with ischemia, accumulated in the mitochondria of the cardiomyocytes dependent on the mitochondrial membrane potential, and binds to the respiratory chain complex V components (including ATP synthesis subunit α, β, the proton channel subunit atp5mc2), which leads to rapidly decrease of the mitochondrial membrane potential, subsequently slowing down of the mitochondrial energy metabolism, preventing the mitochondrial Ca^2+^ overload and inhibition of the mPTP. Thereby, this mitochondrial “quiescent state” reduces cell death from I/R injury. NIR: Near Infrared; OATPs: organic anion transporting polypeptides; IMS: intermembrane space; mPTP: mitochondrial permeability transition pore.

Notably, our findings showed that IR‐780 could not only identify the area at risk of infarction but also more significantly protect cardiomyocytes from I/R‐induced cell death (**Figure** **8**). The mitochondria, which is also the key regulator of both necrosis and apoptosis, are the primary target of IR‐780.^[^ ^4^ ^]^ Of note, the changes in mitochondrial membrane permeability were the most significant events for inducing apoptosis and necrosis. The permeabilization of the outer mitochondrial membrane allows the release of cytochrome c into the cytosol where it functions as a cofactor in apoptosome assembly. The Ca^2+^‐dependent mPTP opening located in the inner mitochondrial membrane mediated mitochondrial necrosis. Notably, IR‐780 could significantly decrease cardiomyocyte necrosis and apoptosis. Consistent with the results of a previous study,^[^ ^4^, ^6^ ^]^ our results indicated that compared with apoptosis, necrosis was the major mode of cell death during I/R. Thus, strategies to block the mPTP opening during I/R were sufficient to inhibit the I/R‐induced cell death. Previously, several drugs (reviewed in ref^[^ ^11^ ^]^) have been reported to inhibit the mPTP opening, which have shown protective roles in animal I/R models. However, the candidate drugs failed to induce a consistent cardioprotective effect in further clinical trials.^[^ ^10^, ^36^ ^]^ Thus, developing novel mPTP‐targeting drugs remains urgently needed. The results of this study showed that mitochondrial respiratory chain complex V (also named F0F1‐ATP synthase) was the primary target of IR‐780, which could induce a rapid mitochondrial membrane potential decrease. Recent studies have reported that respiratory chain complex V is also the key component of the permeability transition pore.^[^ ^11^, ^37^ ^]^ Surprisingly, IR‐780 decreased the mitochondrial membrane potential (evaluated using TMRM, which is also used to reflect mPTP activity^[^ ^38^ ^]^) and conversely increased the calcein fluorescence intensity (another experiment to evaluate mPTP activity^[^ ^39^ ^]^). Moreover, our results showed that ionomycin, a Ca^2+^ ionophore, significantly decreased calcein fluorescence intensity, whereas it had little effects on TMRM intensity. This suggests that the pore response to Ca^2+^ and the pore targeted by IR‐780 are two different channels. Interestingly, these two channels were inhibited by CsA, suggesting that they abut in space. Further analysis of the IR‐780‐targeting components of complex V showed that the ATP synthase c‐subunit was in the list, suggesting that IR‐780 induced the c‐ring opening, dispersed the electron gradient, and more importantly inhibited the Ca^2+^‐regulated mPTP opening. However, the exact interactions of these two pores require further investigation.

In addition to mPTP opening inhibition, IR‐780 also markedly curbed the mitochondrial metabolism, including the TCA cycle, OXPHOS, and even glycolysis, which induced the mitochondria into a relative “quiescent state,” which additionally exhibited a slightly swollen morphology, decreased mitochondrial membrane potential, and ROS production. These properties were also observed in cells with mitoK_ATP_ activation_._^[^ ^40^ ^]^ The activated mitoK_ATP_ channels induce a K^+^ influx into the mitochondrion that caused mild mitochondrial swelling and ROS production.^[^ ^41^ ^]^ The mitoK_ATP_ opening mediates the protective roles of ischemic conditioning and has shown great potential in I/R treatment.^[^ ^3b^ ^]^ Additionally, this “quiescent state” of the mitochondria was shown in drug inducible uncoupling, wherein the low FCCP concentration (100 nm) could induce ROS‐dependent cardioprotection^[^ ^42^ ^]^ although without detectable depolarization.^[^ ^43^ ^]^ Thus, we supposed that the cardioprotection of IR‐780 may be partly mediated by the ROS‐activated intrinsic protection mechanism. However, how IR‐780 induces mitochondrial ROS production remains unclear.

In summary, we have shown for the first time that an NIR heptamethine dye IR‐780 has the potential to rapidly monitor the area at risk following I/R injury by selectively accumulating in the cardiomyocytes of the at‐risk heart tissues. IR‐780 could bind to plasma albumin proteins and be released into the acidic microenvironment of ischemic tissues. The IR‐780 uptake by cardiomyocytes is dependent on OATPs and mitochondrial membrane potential. Moreover, preconditioning with IR‐780 or timely IR‐780 administration before reperfusion significantly reduces ischemia and oxidative stress‐induced cell death, as well as avoids myocardial remodeling and heart failure in both rat and pig models. The protective effects of IR‐780 relies on its binding to the subunits α, β, and the proton channel subunit (atp5mc2) of the mitochondrial OXPHOS electron transport chain complex V, thereby rapidly decreasing the mitochondrial membrane potential and subsequently slowing down the mitochondrial energy metabolism, further inducing the mitochondria into a “quiescent state.” Moreover, IR‐780 binding to complex V resulted in mPTP opening inhibition by preventing mitochondrial Ca^2+^ overload. Our findings identify IR‐780 as a promising injured cardiomyocyte‐targeting agent for both rapid imaging and cardioprotection of I/R injury.

## 4 Experimental Section

### Cell Culture

Rat H9C2 cells were purchased from American Type Culture Collection and cultured at 37 °C and 5% CO_2_ in Dulbecco's Modified Eagle Medium (DMEM) with low NaHCO_3_ (1.5 g L^−1^) containing 10% fetal bovine serum (Gibco) with 100‐U mL^−1^ penicillin and 0.1 mg mL^−1^ streptomycin (Beyotime). The cells of passages 3–8 were used for further experiments. The 4mt‐GCamp6 lentiviral vector was purchased from BrainVTA (Wuhan, China). Transfection of the 4mt‐GCamp6 in H9C2 cells was performed according to the manufacturer's instructions. Following 7‐day culturing, successfully transfected 4mt‐GCamp6‐positive cells were sorted using a Beckman Moflo XDP flow cytometer with 488 nm excitation and 525 nm emission.

### IR‐780 Uptake In Vitro

H9C2 cells were seeded into six‐well plates at a density of 5 × 10^5^ cells and were used for further experiments when up to 90% of confluence was reached. The cells were treated with DMEM with amiloride (75 µg mL^−1^, HY‐B0285, MCE), BSP (250 µm, S0252, Sigma), chlorpromazine (15 µg mL^−1^, 31 679, Sigma), methyl‐β‐cyclodextrin (MβCD, 7.5 mm, 779 776, Sigma), FCCP (10 µm, C2920, Sigma), rotenone (10 µm, 45 656, Sigma), oligomycin A (10 µm, 495 455, Sigma), HSA (1 µm, A1887, Sigma), HSA (1 µm, A1887, Sigma) + ibuprofen (100 µm, I0415, TCI), or ice or hypoxic conditions (5% O_2_) or different pH conditions (5.2, 6.2, 7.4, and 8.9) for 30 min; subsequently, IR‐780 (0.1 µm, 425 311, Sigma) was added into the medium for another 20 min. Next, the cells were washed thrice with PBS and were for further NIR imaging using the Leica NIR fluorescent microscope and flow cytometric analysis using a BD Accuri C6 Flow Cytometer.

### Animals

In this study, 6–10‐week‐old male SD rats weighing ≈250–300 g and 3–4‐month‐old male Bama miniature pigs weighing 28–32 kg were used from the Animal Center of Army Medical University (AMU). In vivo experiments were conducted in accordance with the Guidelines for the Care and Use of Laboratory Animals of the AMU, and all procedures were approved by the Animal Care and Use Committee of the AMU (approval number: AMUWEC2020690).

### SD Rat I/R and Ischemia Model

Rats were anesthetized with pentobarbital (40 mg kg^−1^, i.p.) and ventilated on a Rayward rodent respirator via a tracheostomy. Parasternotomy was performed, and a reversible suture was placed around the left anterior descending (LAD) coronary artery. Myocardial I/R was induced by tightening the suture for 30 min and subsequently loosening it (2 h reperfusion in acute I/R injury or 4‐week reperfusion in chronic I/R experimental setting or without loosening in the ischemia model). The animals were allowed to recover from the surgery.

### Bama Miniature Pig I/R Model

The male Bama miniature pigs were sedated with a cocktail of 4 mg kg^−1^ zolazepam hydrochloride and 4 mg kg^−1^ tiletamine hydrochloride injected intramuscularly. A mixture of 1%–2% isoflurane dissolved in 40% air and 60% oxygen was continuously administered for inhalatory anesthesia via a Rayward respiratory anesthesia machine for large animals. Electrocardiogram, heart rate, and arterial pressure were constantly monitored. Thoracotomy was performed in the left fourth intercostal space; subsequently, the pericardial sac was opened to expose the heart. Myocardial I/R was induced by coronary occlusion. The LAD coronary artery was encircled by a suture thread; subsequently, the suture was tightened to occlude the vessel, which was confirmed by the presence of regional myocardial cyanosis (Figure 1A, Supporting Information). Moreover, vessel occlusion was confirmed by NIR imaging, wherein IR‐780 (0.667 mg kg^−1^) was intravenously injected immediately following occlusion. Ten minutes later, the hearts were harvested and sectioned through four horizontal planes. The sections were subsequently photographed using an NIR imaging instrument, and NIR signals were absent in the regions supplied by the LAD coronary artery ( Figure 1A, Supporting Information). Myocardial I/R was induced by tightening the suture for 90 min and subsequently removed it to start the reperfusion phase.

### Infarct Size Quantification

Briefly, following the 2 h reperfusion, the LAD coronary artery was re‐occluded at the same position as the original infarction. To delineate the area at risk, the left ventricle was injected with 1 mL of 1% EBD. The heart was then excised and cut into four transverse slices. To differentiate infarction from viable tissues, the slices were subsequently incubated with fresh 1% TTC at 37 °C for 15 min. Next, the slices were photographed, and the regions of the left ventricle, which were negative for EBD staining and positive for TTC, were calculated.

### IR‐780‐Based Imaging

In the SD rat I/R or ischemia model, myocardial infarction was induced in the male SD rats as previously described; immediately following ischemia or reperfusion, they received intraperitoneal injection of IR‐780 (0.667 mg kg^−1^) or HSA‐IR‐780 (containing the equivalent dose of IR‐780 [0.667 mg kg^−1^]). The hearts were harvested at indicated timepoints following reperfusion or ischemia. NIR imaging was subsequently performed on the main organs including liver, lung, spleen, kidney, muscle, intestine, the whole heart and transverse slices using a mobile NIR image scope (developed by Professor Wang Ping from Huazhong University of Science and Technology) or an in vivo imaging system (VISQUE Smart‐LF, Vieworks). For the BSP blocking experiment, animals received direct intramyocardial injections of 200‐uL BSP (0.25 mg kg^−1^) or PBS in the LV anterior wall 5 min before I/R. To compare the area at risk by Evans blue and IR‐780 and infarct size by TTC and IR‐780, 8 heart transverse slices of 2 h post reperfusion were selected, and the following areas were analyzed by Image J software: the area at risk of Evans blue negative (A), infarct size of TTC (B) and the whole heart area (C) in the photographs, and the IR‐780 accumulated area (D) and the whole heart area (E) in the NIR images. The comparison (F) of the area at risk by Evans blue and IR‐780 were calculated as follows: F = (D/E)/(A/C) × 100. The comparison (G) of infarct size by TTC and IR‐780 were calculated as follows: G = (B/E)/(A/C) × 100.

### IR‐780‐Based Protection

The in vivo IR‐780 precondition group consisted of rats or pigs that received IR‐780 (0.667 mg kg^−1^ for rats and pigs) intraperitoneal injection once daily for 3 days before I/R or ischemia. In the chronic I/R models, rats or pigs were administered another IR‐780 (0.667 mg kg^−1^) once a week. The in vivo IR‐780 treatment group comprised rats or pigs that received IR‐780 (0.667 mg kg^−1^ for rats and pigs) intraperitoneal injection immediately following ischemia. In the chronic I/R models, rats or pigs were administered another IR‐780 (0.667 mg kg^−1^) once a week. The control group included rats or pigs that received an equivalent volume of PBS intraperitoneal injection once daily for 3 days before I/R or ischemia. In the chronic I/R models, rats or pigs received another equivalent volume of PBS injection once a week. The whole blood and serum samples of the rats or pigs at 1 month following reperfusion in the chronic I/R models were seeded for blood routine, liver function, kidney function analysis.

For the in vitro H9C2 cell preconditioning, the cells were treated with IR‐780 (0.1 µm in DMEM) at 37 °C for 20 min; subsequently, IR‐780 was washed out. After 24 h, the cells were used for subsequent experiments. For the in vitro H9C2 cell treatment, the cells were treated with IR‐780 (0.1 µm in DMEM) at 37 °C for 20 min; subsequently, IR‐780 was washed out for succeeding experiments. For the H9C2 cell control, the cells were treated with an equivalent volume of DMEM at 37 °C for 20 min and subsequently changed to full medium (DMEM with 10% FBS) for subsequent experiments.

### Statistical Analysis

All statistical analyses were performed using Statistical Package for the Social Sciences (version 13, SPSS Inc., Chicago, IL, USA). Data were expressed as means ± SD. Data of two groups were compared using two‐sided unpaired Student's t‐test. Multiple group comparisons were performed using one‐way analysis of variance. Analysis of variances were followed by LSD post hoc tests for multiple comparisons when the normality and homogeneity of the variance assumptions were satisfied, while Tamhane with heterogeneous variance. A *p*‐value <0.05 was considered statistically significant. All experimental *n* numbers are provided in the figure legends.

## Conflict of Interest

The authors declare no conflict of interest.

## Author Contributions

Z.C., X.T., T.J. contributed equally to this work. Z.C. contributed to conception and design, data analysis and interpretation, financial support, manuscript writing; X.T., T.J., Y.W. contributed to provision of study material, collection and assembly of data, data analysis and interpretation; L.D., C.S., J.W., X.Y., H.L. contributed to thoracotomy of the pigs; G.S., C.Z., L.Q. contributed to collection and assembly of data; L.L., X.C. contributed to chemical synthesis. C.S. contributed to conception and design, financial support, final approval of manuscript.

## Supporting information

Supporting Information

Supplemental Video 1

## Data Availability

The data that support the findings of this study are available from the corresponding author upon reasonable request.
