# Supplementary material for: Pharmaceutical Manipulation of Mitochondrial F0F1‐ATP Synthase Enables Imaging and Protection of Myocardial Ischemia/Reperfusion Injury Through Stress‐induced Selective Enrichment
Source: Adv Sci (Weinh). 2023 Dec 14;11(9):2307880. doi: 10.1002/advs.202307880 (PMC10916578; doi:10.1002/advs.202307880)
Supplement: Supplementary file 1 — Supporting Information [file ADVS-11-2307880-s001.pdf]

ADVANCED SCIENCE
Open Access

Supporting Information

for *Adv. Sci.*, DOI 10.1002/advs.202307880

Pharmaceutical Manipulation of Mitochondrial F0F1-ATP Synthase Enables Imaging and Protection of Myocardial Ischemia/Reperfusion Injury Through Stress-induced Selective Enrichment

*Zelin Chen, Xu Tan, Taotao Jin, Yu Wang, Linyong Dai, Gufang Shen, Can Zhang, Langfan Qu, Lei Long, Chongxing Shen, Xiaohui Cao, Jianwu Wang, Huijuan Li, Xiaofeng Yue and Chunmeng Shi**

Supporting Information

Pharmaceutical Manipulation of Mitochondrial F0F1-ATP Synthase Enables Imaging and Protection of Myocardial Ischemia/Reperfusion Injury Through Stress-induced Selective Enrichment

Zelin Chen, Xu Tan, Taotao Jin, Yu Wang, Linyong Dai, Gufang Shen, Can Zhang, Langfan Qu, Lei Long, Chongxing Shen, Xiaohui Cao, Jianwu Wang, Huijuan Li, Xiaofeng Yue, Chunmeng Shi*

Supporting methods

IR-780 and HSA binding assessment Firstly, IR-780 was dissolved in PBS or1μM HSA solution to give a final concentration of 1μM for emission spectrum analysis and scanning the wavelength from 750 to 900 nm following the excitation at 740 nm. Later, a protein-binding assay was conducted. Briefly, warfarin, ibuprofen, digoxin and quinidine (all from TCI) were used as competitive inhibitors of albumin-binding sites I, II, III and α1-glycolipoprotein respectively. The buffer solution containing 5% DMSO and 1μM HSA were pre-incubated with or without these inhibitors (100 μM) for 30 min at room temperature. Immediately after the addition of IR-780 at a concentration of 1μM, the fluorescence intensity was measured following the excitation at 740 nm and the emission from 750 to 900 nm. Last, the specific release of IR-780 from HSA was investigated by titrating with HCL solution. The changes of absorption characteristic spectra of HAS-IR-780 complexes were detected with the decrease of pH values from 7.22 to 6.04.

Preparation and Characterization of HSA-IR-780 5 mg IR-780 was firstly dispersed in 1 ml methanol. HSA (Sigma-Aldrich, 200 mg) and 10 μL TEA (triethylamine) were mixed in 10 ml

ultrapure water for 30 min. Then, the mixture was added with 1 ml above-mentioned methanol solution of IR-780 for overnight vigorous stirring. After 24 h dialysis of the reaction solution with a dialysis bag (MWCO 10 KDa), HSA-IR-780 nanoparticles were obtained.

LDH release assay Hearts of control, IR-780 treatment, IR-780 precondition groups were processed with I/R, 2 hours later, the blood were harvested with promoting coagulating tubes. The blood samples were then centrifugated with 3000g, 20min, the plasma was collected for further LDH release assay with a LDH release assay kit (ab65393, Abcam) according to the manufacturer's instructions.

Cell membrane permeability to EBD EBD (10 mg ml^{-1}) was intraperitoneally injected into rats (100 μg g^{-1} body weight) of control, IR-780 precondition, IR-780 treatment groups. 24 hours later, the rats were subjected to 30 min ischemia and 2 hours reperfusion. The rats were then sacrificed, and the injured hearts was harvested and embedded in optimal cutting temperature (OCT) compound, snap frozen in -80℃ and cut into 10 μm cryosections. The cryosections were then fixed with 4% Polyformaldehyde for 10min, then washed with PBS three times and mounted with Prolong Diamond antifade Reagent containing DAPI. Then the EBD signals were captured in a full slide scanning system (VS200, Olympus). The regions of the left ventricle, EBD positive area were calculated by image J.

Echocardiography 4 weeks after I/R, the M-mode and two-dimensional echocardiography was performed using an ultrahigh resolution small animal imaging system (VEVO 2100; Visual Sonics, Toronto, Canada) equipped with a linear array probe (MS400, Visual Sonics) with a frequency of 24 MHz to acquire conventional transthoracic echocardiography images for serially assessment of rat cardiac structure and function. 4 weeks after I/R, the

echocardiography of pigs was measured with a clinical ultrasonic diagnostic instrument (E3EXP, Sonoscape Medical Corp.)

Isolation of mitochondria Mitochondria were isolated from the H9C2 cells using the Mitochondria Isolation Kit (37612， Qproteome) according to the manufacturer protocol. Briefly， 5 x 10^6 to 2 x 10^7 cells were centrifuged at 500 g for 10min at 4°C, then discard the supernatant. The cells were then incubated with Lysis Buffer for 10 min at 4°C, then centrifuge the lysate at 1000 g for 10 min at 4°C, and the cell pellets were resuspended with ice-cold Disruption Buffer, and then centrifuge the lysate at 1000 g for 10 min at 4°C, the supernatant were further centrifuged at 6000 g for 10 min at 4°C. The mitochondrial pellets were washed with Mitochondria Storage Buffer and then centrifuged at 6000 g for 20 min at 4°C. The mitochondrial pellets were resuspended with Mitochondria Storage Buffer for further experiments.

Isolation of adult rat cardiomyocyte Adult rat cardiomyocytes were isolated from SD rats, following previous protocol. [1] Briefly, a rat was injected with 300 units of heparin (i.p.). 15 min later, animals were anesthetized by intraperitoneal injection of 100 mg/kg pentobarbital. The heart with lungs and thymus attached was quickly removed and put into the ice-cold buffer with EDTA. Then, tied the aorta and mounted on a modified Langendorff perfusion system. Subsequently, the heart was perfused with 37 °C oxygenated Krebs-Henseleit Buffer (KHB) solution plus calcium (200 μM), and then perfused with 0.5mg/mL collagenase II and 0.5mg/mL collagenase IV (Worthington) and 0.05 mg/mL protease (Sigma) for 30 min. Afterward, the heart was cut into small pieces for further digestion under gentle agitation in the enzyme solution. Rod-shaped adult cardiac myocytes were collected by centrifugation and

followed by the gradual addition of CaCl2. Freshly isolated cardiac myocytes were plated on dishes pre-coated with 40 μg ml^{-1} laminin for 2 h. All myocytes were cultured in M199 medium (Sigma) supplemented with 10 mM glutathione, 5 mM taurine, at 37 °C and 5% CO2 in a humidified incubator.

Mitochondrial ETC complex activity The mitochondria complete OXPHOS activity assay kit (ab110419, Abcam) was used to test the mitochondria ETC complex activity treated with different IR-780 concentration according to the manufacturer protocol respectively. Briefly, the detergent-solubilized mitochondria were added with different concentration of IR-780. The complex I activity were tested by measuring the rate of oxidation of NADH to NAD+ (reflected with the absorbance (OD340nm)) in kinetic mode. Complex II were tested by measuring the rate of production of the ubiquinol (reflected with the absorbance (OD600nm)) in kinetic mode. Complex III were tested by measuring the conversion of oxidized cytochrome c into reduced form (reflected with the absorbance (OD550 nm)). Complex IV were tested by monitoring the decrease in absorbance at OD550 nm. The conversion of ADP to ATP coupled to the oxidation of NADH to NAD^+. Thus, complex V were tested by measuring the decrease in absorbance (OD340 nm). All the measurement was in a Thermo Fisher Varioskan LUX microplate reader.

Mitochondrial oxygen consumption The mitochondrial oxygen consumption rate (OCR) was measured using an XF24 Extracellular Flux Analyzer (Seahorse Biosciences) according to manufacturer protocol. Normal H9C2 cells or H9C2 cells 72 hours following IR-780 (0.1 μM) treated were plated at a density of 10,000 cells per well on XF24 culture plates. Oxygen concentration in plates was measured at basal conditions and after sequential addition of compounds as indicated in corresponding figure legends. Concentration of compounds used

were: IR-780 (0.1, 0.5, 1 μM); oligomycin A (1.5 μM); FCCP (3 μM); and a mixture of rotenone (500 nM) and antimycin A (500 nM). A minimum of three wells were utilized per condition to calculate OCR.

ROS Measurements Mitochondrial ROS production in H9C2 cells or freshly isolated rat cardiomyocytes with IR-780 (0.1 μM) for 20min or 24h, hypoxia (5% O_2) 2h with or without preincubation of IR-780 (0.1 μM) for 20min, or hypoxia hypoxia (5% O_2) 2h then reperfusion 5 min with or without preincubation of IR-780 (0.1 μM) for 20min, Mito tracker-green (0.25 μM) for 20 min was measured with MitoSOX Red (M36008, Invitrogen), and total cell ROS production were measured with DCFH-DA (S0033S, Beyontine) according to the manufacturer protocols. Briefly, cells were incubated with Mito SOX Red (1:5000) or DCFH-DA (1:1000) at 37°C for 15 min in the dark, then the cells were collected for measurement of the fluorescent dye by a BD Accuri™ C6 Flow Cytometer or fluorescence images capturing by a spinning disk confocal super resolution microscope (Olympus, SpinSR10).

Histology To detected the localization of the IR-780 in the I/R regions, the IR-780 administrated (the same as described in IR-780 based imaging) hearts at 2 hours following I/R were harvested, and the injured regions were processed as cryosections (10-15 μm). To avoid to get rid of the IR-780, the sections were treated with Triton-100 (0.3%) for only 3-5 minutes, and then staining with incubated overnight at 4°C with anti-α-actin (ab250775, Abcam) diluted in blocking buffer. The next day, primary antibodies were washed thrice in PBS containing 0.3% Triton X100 for 10 min and incubated with Alexa Fluor 488-conjugated secondary antibody (diluted in PBS) for 30 min at 37 °C. The secondary antibodies were then washed away in PBS for 10 min. Slides were mounted with Prolong Diamond antifade Reagent containing DAPI

and then detected the signals of IR-780, α-actin and DAPI with a laser confocal microscope. To detected the subcellular localization of the IR-780 or B-IR-780 in the cells, H9C2 cells were incubated with mito-tracker green (M7514, Invitrogen) for 30 min, and then labeled with IR-780 (1 μM) or B-IR-780 (5 μM) for another 20 min, and then staining with the hoechst for 10 min. finally, the images were captured in a laser confocal microscope. H&E staining for the I/R induced heart tissues: the tissues were processed with normalized dehydration and paraffin embedding protocols, and dewaxed paraffin sections were firstly staining with hematoxylin for 1-2min at room temperature, then incubated in eosin buffer for 1min at room temperature. Successively transfer the slides into staining jars with 80% ethanol for 20 sec, 90% ethanol for 20 sec, 100% ethanol for 1 min and xylene for 3 min. The images were captured in a microscope. The analysis of images was performed by image J.

In vitro I/R injury to cells H9C2 cells were cultured in complete culture media containing DMEM with 10%FBS and 4mM glutamine. Cells were treated with IR-780 (0.1 μM) for 20 min at 37 °C. 24 hours later (precondition group) or immediately following the IR-780 (0.1 μM) treatment (treatment group), the culture media was replaced with hypoxic buffer (DMEM+ 10 mM HEPES, pH=6.5). The plates were put into a hypoxia chamber for 2 hours, then the cells were removed from the hypoxia chamber (5% O_2) and replaced with the normoxia buffer (DMEM+ 10 mM glucose+10mM HEPES, pH =7.4). 1.5 hours later, we measured the cell death with a lactate dehydrogenase (LDH) release assay (ab65393, Abcam) according to the manufacturer's instructions.

Transmission electron microscopy H9C2 cells were harvested at different time points lowing IR-780 treatment. The heart tissues supplied by left anterior descending coronary artery in 2

hours following I/R or 4 hours following ischemia of different IR-780 treatment groups were harvested and cut into 1×1 mm sizes. The cell and tissue samples were fixed with 2.5% glutaraldehyde at 4°C overnight. Samples were stained with 1% osmium acid, dehydrated with a gradient of acetone. The samples were then embedded in Epon812 epoxy resin, and then cut into 80 nm thick ultrathin sections with an ultramicrotome. The 80 nm ultrathin sections were stained with uranyl acetate and lead citrate, and then observed by TME.

TUNEL staining TUNEL staining of the injured heat tissues harvested from different I/R groups described above was performed using in situ cell death detection kit (11684795910, Roche) according to the manufacturer's protocol. Minimum three heart samples in each group were analyzed by counting positive cells per high-power field.

Apoptosis assay H9C2 cells in control, IR-780 precondition and IR-780 treatment groups (described in IR-780 based protection) were treated with H_2O_2 for 2 hours, then cells were harvested, washed twice with cold PBS, resuspended in Annexin-V binding buffer (10 mM HEPES, 150 mM NaCl, 5 mM KCl, 1 mM MgCl2, and 1.8 mM CaCl2, Ph=7.4) with FITC-conjugated Annexin-V (5ul vol. per test, BD Biosciences, 51-65874X) and PI (50 µg/ml, Sigma, P4170) for 20 minutes at room temperature in the dark. Samples were analyzed by BD Accuri™ C6 Flow Cytometer.

Mitochondrial membrane potential ($\Delta\Psi_m$) measurement The measurement of $\Delta\Psi_m$ is based on the distribution of the mitochondrion-selective lipofilic cation dye TMRM (I34361, Invitrogen). H9C2 Cells or freshly isolated adult rat cardiomyocytes with different treatments were loaded with TMRM (1:1000) for 30 min at 37 °C. The cells were harvested for further analysis by BD Accuri™ C6 Flow Cytometer. To capture the image of the TMRM staining, cells loaded with

TMRM were then further incubated with Hoechst 33342 for another 10 min before image capture with a fluorescence microscope.

Cell viability analysis H9C2 cells were seeded in the 96-wells plates (1×10^4 cells $well^{-1}$) and cultured overnight. Cells were then subjected to different concentrations of IR-780 (0, 0.001, 0.01, 0.1, 1, 5 μM) or B-IR-780 (0, 0.01, 0.1, 1, 10, 50 μM) at 37°C for 20 min. 24 hours later, cell viability was assessed using the CCK-8 assay as previously described.[2]

Proximity ligation assay (PLA) H9C2 cells were pretreated with B-IR-780 (5 μM) or biotin (5 μM) for 20min at 37 °C were fixed with 4 % paraformaldehyde and blocked with the blocking solution supplied by the Duolink PLA kit (DUO96020, sigma) according to the manufacturer's instructions. Briefly, the fixed cells were treated with Triton-100 (0.1%) for 5 min, then incubated with anti-biotin antibody (ab201341 or ab53494, Abcam) and anti-Atp5f1a antibody (ab14748, Abcam), anti- Atp5f1b antibody (MA1-930, Invitrogen), anti- Atp5f1d antibody (PA5-80362, Invitrogen), anti-Atp5mc2 antibody (PA5-114397, Invitrogen), anti-Atp5f1c antibody (PA5-29975, Invitrogen), anti-Atp5po antibody (PA5-96932, Invitrogen) overnight. After multiple washings, cells were incubated successively with PLA probes, ligation solution and amplification solution at 37 °C. Cover-slips were mounted and the images were examined using a full slide scanning system (VS200, Olympus) to capture the NIR signal and green signal of the PLA.

Non-targeted metabolomics The H9C2 cells were treated with IR-780 (0.1 μM) at 37 °C for 20 min, then the cells were harvested at 20min, 6 hours, 24 hours, 72 hours following IR-780 administration. For non-targeted metabolomics analysis, 5×10^6 H9C2 cells per sample of 6 replicates in each time point was performed using a quadrupole time-of-flight mass

spectrometer (Sciex TripleTOF 6600) coupled to hydrophilic interaction chromatography via electrospray ionization in Shanghai Applied Protein Technology Co., Ltd according their standard detection and data analysis protocols.

Biotin-IR-780 pull-down assay and mass spectrometry For pulldown assays, cells were treated with Biotin (5 μM) or Biotin-IR-780 (5 μM) for 20 min. After treatments, the mitochondria were isolated as described above. The mitochondria pellets were lysed for 20 min in the lysis buffer (0.025 M Tris, 0.15 M NaCl, 0.001 M EDTA, 1% NP-40, 5% glycerol, pH 7.4) with protease inhibitor cocktail (Millipore) on ice. The mitochondrial or the whole cell lysates were then spun at 13,000 g for 10 minutes at 4 °C. Supernatant was transferred to a new tube and protein concentration was measured. 1,000 μg of the lysate at 1 μg μL^{-1} was then used to incubate with 40 μL Streptavidin Sepharose (3419S, Cell Signaling Technology) for at least 4 hours. Then the sepharose was then spun and washed for 3 times. Binding proteins were eluted by with elution buffer (pH 2.8, contains primary amine). The pulldown elution was then used for protein SDS gel running and then stained with Coomassie blue or western blot as described previously(*54*) (the primary antibodies were as follows: anti-biotin antibody (ab201341, Abcam) and anti-atp5f1a antibody (ab14748, Abcam), anti-atp5f1b antibody (MA1-930, Invitrogen), anti- Atp5f1d antibody (PA5-80362, Invitrogen), anti-Atp5mc2 antibody (PA5-114397, Invitrogen), anti-Atp5f1c antibody (PA5-29975, Invitrogen), anti-Atp5po antibody (PA5-96932, Invitrogen) or Mass Spectrometry. The whole elution of each mitochondrial pulldown sample was used for Mass Spectrometry. The Mass Spectrometry analysis service was provided by Shanghai Applied Protein Technology Co., Ltd.

Synthesis of Biotin-IR-780

Synthesis of **1**

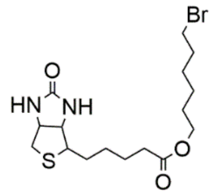

Biotin (20 mmol), 6-bromohexanol (40 mmol) and p-methylbenzenesulfonic acid (20 mmol) were dissolved in toluene (150 mL). The reaction kept refluxing for 14-16h at 110℃. After that, the solvent was evaporated to dryness. The crude product was purified by silica gel chromatography using CH_2Cl_2/CH_3OH(v/v=40:1~30:1) as the eluent to obtain compound **1** as a white solid. They were characterized by both ^{1}H NMR spectra, ^{13}C NMR and high-resolution mass spectra (HRMS), which are shown as following：

^{1}H NMR (600 MHz, DMSO) δ 6.39, 6.32, 4.26, 4.26, 4.25, 4.24, 4.09, 4.08, 4.08, 4.07, 3.96, 3.95, 3.94, 3.49, 3.48, 3.46, 3.06, 3.05, 3.04, 3.04, 3.03, 2.78, 2.78, 2.76, 2.76, 2.54, 2.52, 2.45, 2.25, 2.24, 2.23, 1.77, 1.76, 1.75, 1.73, 1.72, 1.54, 1.51, 1.50, 1.49, 1.36, 1.34, 1.33, 1.32, 1.30, 1.28, 1.27, 1.26. ^{13}C NMR (151 MHz, DMSO) δ 172.94, 162.74, 63.63, 61.09, 59.21, 55.42, 35.17, 33.39, 32.14, 28.07, 28.04, 28.02, 27.20, 24.60, 24.58. MS[M]: calc. 406.09, measured 407.09.

Synthesis of **2**

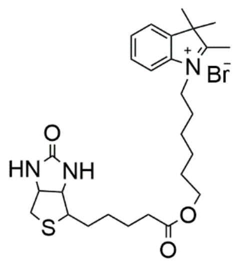

Under nitrogen atmosphere, **1**(6 mmol) and 2, 3, 3-trimethylindolenine (7.2 mmol, 1.2eq) were dissolved in 25mL of toluene. Then, triethyamine (12 mmol, 2eq) was injected dropwise while

stirring and reacted at 110°C for 10-12h. The mixture washed by diethyl ether. After a laying process, supernatant liquor was poured, following the volatilization of diethyl ether, the residue oil (orange to pink) can be used in the next step without further purification.

Synthesis of **3**

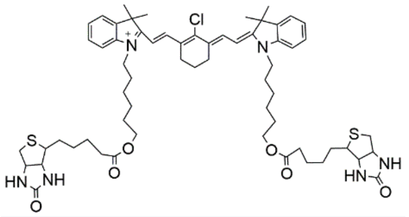

The crude product **2** from last step was used directly in this reaction. Acetic anhydride (20 mL) and sodium acetate (6 mmol) were added into two-neck flask containing **2** (6 mmol) and (E)-2-chloro-3-(hydroxymethylene) cyclohexane-1-carbaldehyde (3 mmol) under nitrogen atmosphere. The solution was stirred at room temperature for 1h. Then the solution was washed by diethyl ether. After the volatilization of diethyl ether, the solid was purified by silica gel chromatography using CH_2Cl_2/CH_3OH(v/v=20:1~10:1) as the eluent to obtain compound **3** as a brown solid. They were characterized by both ^{1}H NMR spectra, ^{13}C NMR and high-resolution mass spectra (HRMS), which are shown as following:

^{1}H NMR (600 MHz, DMSO) δ 9.08, 8.19, 8.17, 7.57, 7.56, 7.39, 7.38, 7.37, 7.36, 7.35, 7.23, 7.22, 7.21, 6.36, 6.30, 6.26, 6.24, 4.22, 4.21, 4.20, 4.15, 4.04, 3.93, 3.92, 3.91, 3.05, 3.03, 3.03, 3.02, 3.01, 3.00, 2.73, 2.72, 2.71, 2.70, 2.63, 2.49, 2.20, 2.19, 2.17, 1.59, 1.48, 1.34, 1.31, 1.29, 1.13, 1.11, 1.10. ^{13}C NMR (151 MHz, DMSO) δ 172.95, 172.27, 162.77, 148.03, 143.03, 143.01, 142.49, 142.12, 141.12, 126.17, 125.26, 122.62, 122.60, 109.80, 109.78, 109.62, 109.61, 101.66, 63.65, 61.10, 59.24, 55.44, 49.07, 45.74, 43.78, 39.24, 33.36, 28.09, 28.07, 28.04, 27.54, 26.95, 25.90, 25.78, 25.20, 24.60. MS[M-Br]: calc. 1107.56, measured 1107.555.

Calcein release assay H9C2 cells or freshly isolated adult rat cardiomyocytes or freshly isolated mitochondria received different treatment including IR-780 (0.1 μM or 0.5 μM) for 20 min, Cyclosporin A (CsA, 10 μM, C-093, Sigma) 30min and IR-780 (0.1μ M or 0.5 μM) for another 20 min, Ionomycin (1 μM) for 10min, or Mito tracker-red (0.25 μM, M22425, Invitrogen) for 20 min, and calcein release assay was carried out according to manufacturer's instructions in Transition Pore Assay Kit (I35103，Ivitrogen). Briefly, cells of different treatments were washed twice with the modified HBSS buffer, and then incubated with sufficient labeling solution (1 mM calcein and 1 mM CoCl2) for 15 min at 37 °C. After incubation, cells were washed with warm HBSS buffer and analyzed by a flow cytometry BD Accuri C6 or observed in a Leica laser confocal microscope. To constantly monitor the effects of IR-780 on the mPTP, the H9C2 cells were seeded in a 96 wells plates. 24 hours later the cells were treated with IR-780 for 20min at 37 °C, and then incubated with the labeling solution for 15 min at 37 °C, then the fluorescence signals were constantly detected for 6 times (5 second per time) in a Thermo Fisher Varioskan LUX microplate reader. Next, the Ionomycin (1 μM) were added into each well, and the fluorescence signals were constantly detected for another 170 times (5 second per time). And the slopes of fluorescent decrease were calculated. To detected the effects of Ca^{2+} on the mitochondrial membrane potential, H9C2 cells were treatment with Ionomycin (1 μM) for 10min and then incubated with sufficient labeling solution (1 mM calcein and 1 mM CoCl2) for 15 min at 37 °C, next incubated with TMRM (1:1000, I34361, Invitrogen) for another 30 min. The cells were observed in a fluorescence microscope.

Optical measurements of Ca^{2+} in single cells The H9C2 cells expressing 4mt-GCamp6 (4mt-

GCamp6 Lentivirus were purchased from BrainVTA Co.) were seeded in a 20 mm glass button dishes. The detection dishes containing adherent cells was washed three times with fresh DMEM medium immediately prior to measurements. Single cell detections were performed on an inverted microscope platform (Olympus, IX71) in a Faraday cage with all room lights switched off at room temperature (23 °C). The optical nanoprobe (Raymete) with the tip diameter about 590 nm was precisely positioned with a nanomanipulator (0.5 μm precision) at fixed locations on the cell membrane of an isolated cell. The fluorescence signal of the 4mt-GCamp6 (Excitation at 488 nm and emission 525 nm) upon 1μM ionomycin stimulation with or without pre-IR-780 (0.1μM, 15 minutes before) was recorded in real-time by the fluorescence detection system assembled as reported.[3]

Fluorescence detection of Ca^{2+} by microscope The H9C2 cells expressing 4mt-GCamp6 were seeded in a 20 mm glass button dishes. The next day, cells were treated with IR-780 (0.1 μM) for 15 min, then washed with fresh DMEM medium for three times. The fluorescence images of the 4mt-GCamp6 (Excitation at 488 nm and emission 525 nm) of control and IR-780 pre-treatment groups upon 1μM ionomycin stimulation were captured by a spinning disk confocal super resolution microscope (Olympus, SpinSR10) in a real-time model. The recorded images were analyzed and quantified using the cellSens Dmension software. A total of random selected 20 cells in each group were analyzed.

For image analysis, background intensity was performed to choose the intensity of a nearby cell-free region from the signal of the imaged cells. F0 is calculated as the initial background-subtracted fluorescence intensity. F is the background-subtracted fluorescence intensity at each time point.

For Ca2+ measurements in adult rat cardiomyocytes, the freshly isolated adult rat cardiomyocytes were seeded in a laminin precoated 20 mm glass button dishes. Then, the cardiomyocytes were treated with IR-780 (0.1 μM) for 20 min or IR-780 (0.1 μM) 20min and Ionomycin (1 μM) for another 10 min or Ionomycin (1 μM) for 10min. then the cells were incubated with Rhod-2 AM reduced by sodium borohydride (1:1000, Invitrogen) for 20 min at 37 °C. The cells were then observed in a fluorescence microscope. At least 10 images were selected in each group to calculate the fluorescence intensity of the cells by Image J.

Supporting Figures and Figure legends

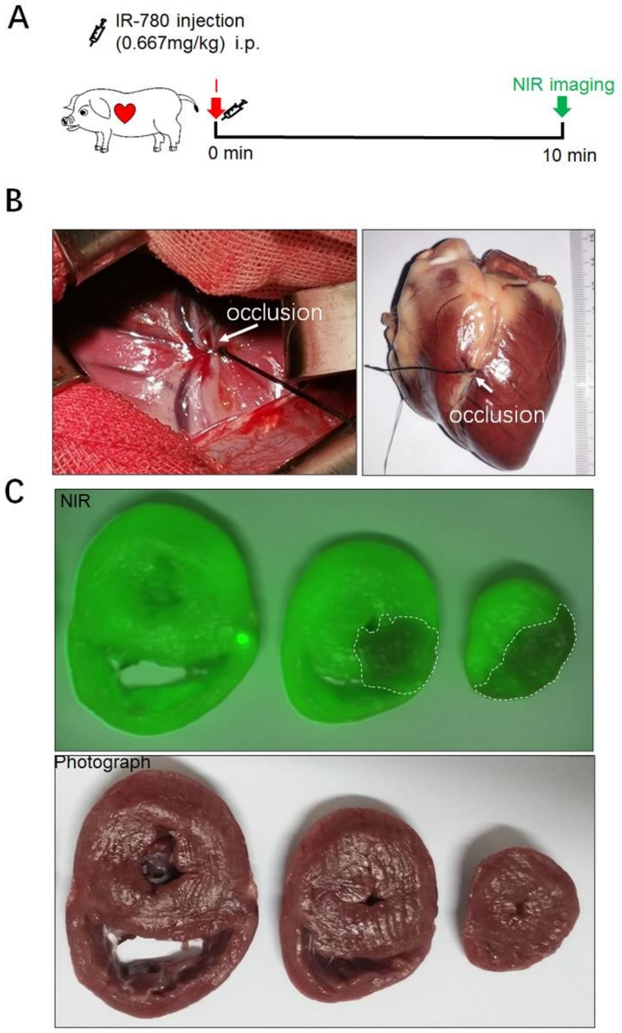

Figure S1. IR-780 could act as a fluorescent indicator for in vivo imaging. A) Schematic of the IR-780 based imaging. B) left: Representative photograph taken during pig surgery to show the occluded site of the LAD. Right: Representative photograph of the harvested pig heart following the occluding of the LAD. C) Representative NIR imaging (upper panel) and the corresponding bright-field photograph (lower panel) of the transverse sections.

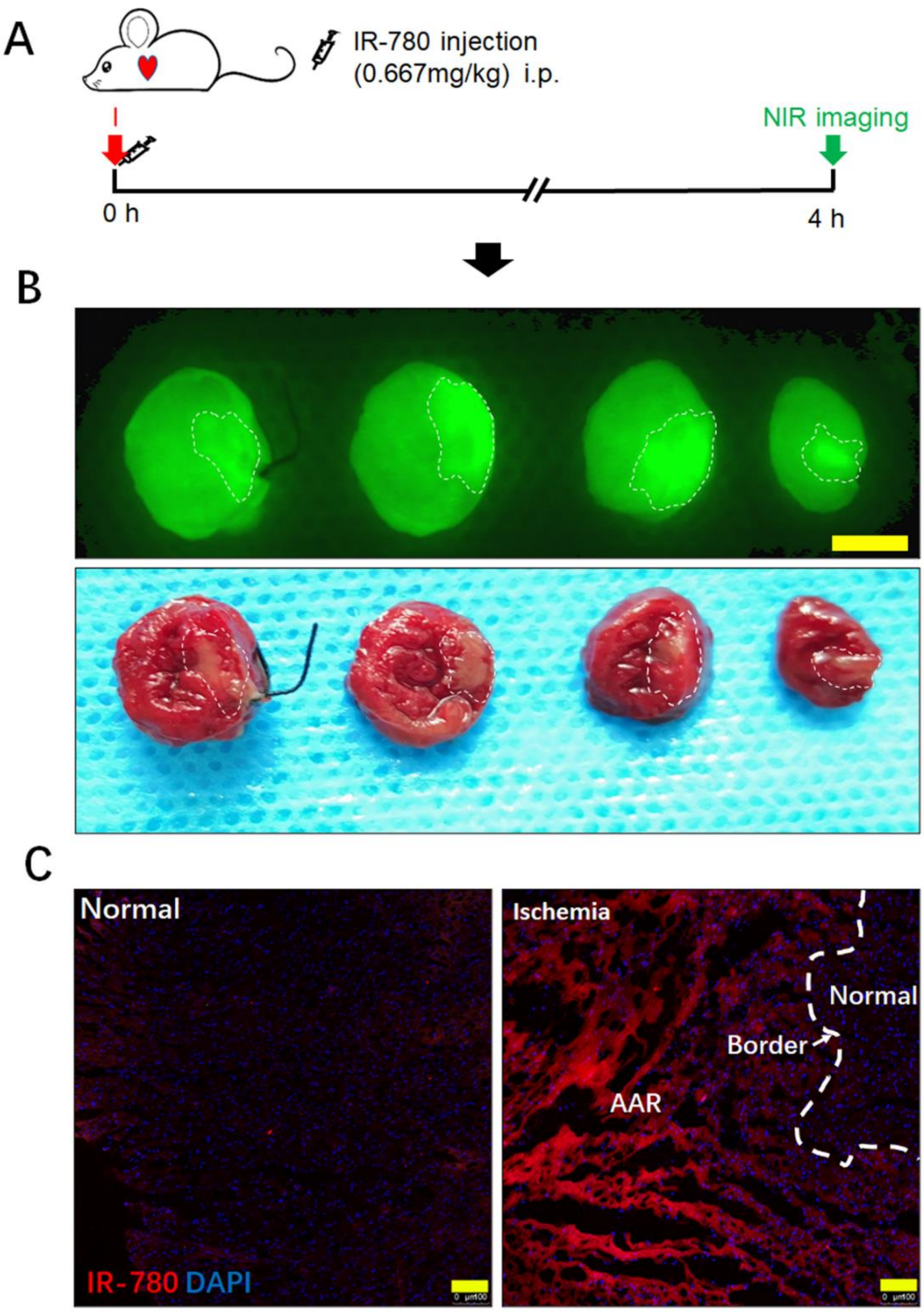

Figure S2. IR-780 accumulates in the infarcted heart tissues in rat ischemia models. A) Schematic of the IR-780 based imaging protocols for ischemia models. B) Upper panel: Representative NIR photographs of the transverse sections of the rat heart 4 h following ischemia. lower panel: the corresponding bright-field photographs. Scale bar: 5 mm. C) Representative confocal images of the normal and the injured rat heart tissues 4 h following ischemia. Scale bar:100 μm.

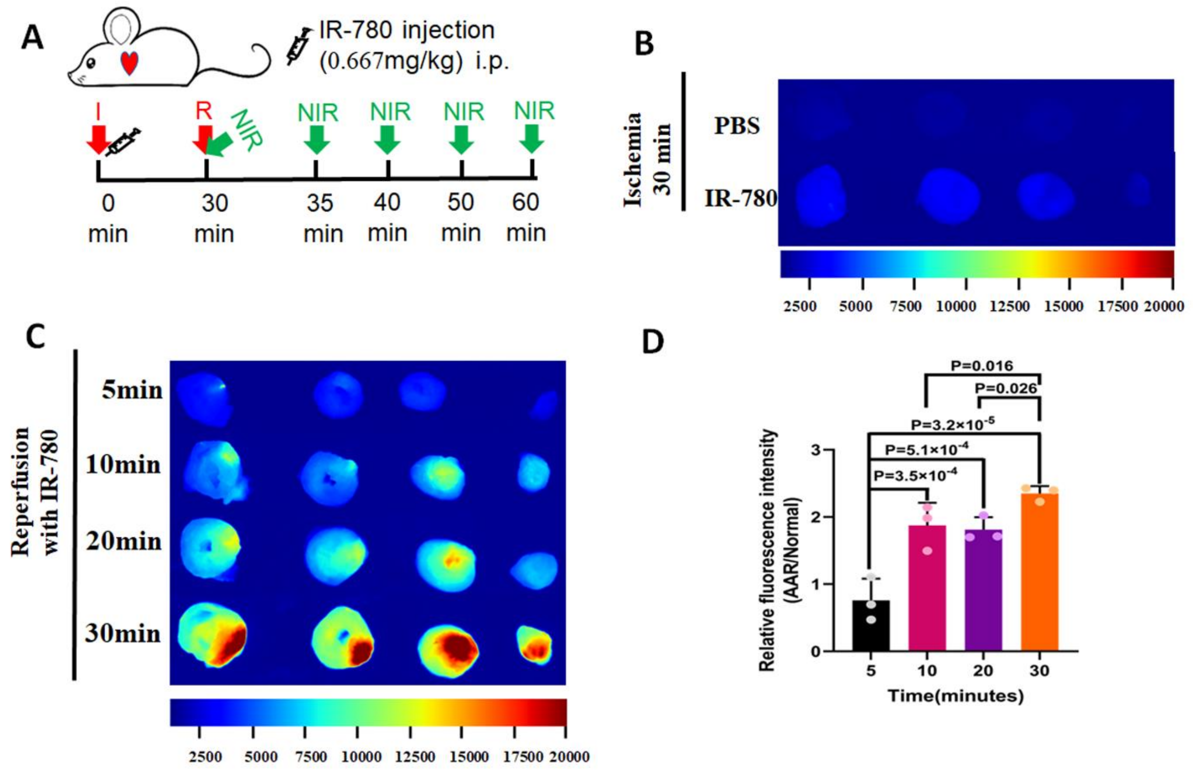

Figure S3. The dynamic accumulation of IR-780 in the injured heart tissues. A) Schematic of the detecting protocols for IR-780 administrated immediately after ischemia in rat I/R models. B) Representative images of the NIR fluorescent signals of the heart tissues treated with IR-780 or PBS 30 minutes following ischemia. C, D) Representative images and quantitative data of the NIR fluorescent signals of the heart tissues treated with IR-780 at indicated time points following reperfusion (n=3 per time point). All the *p* values are present in the graphs.

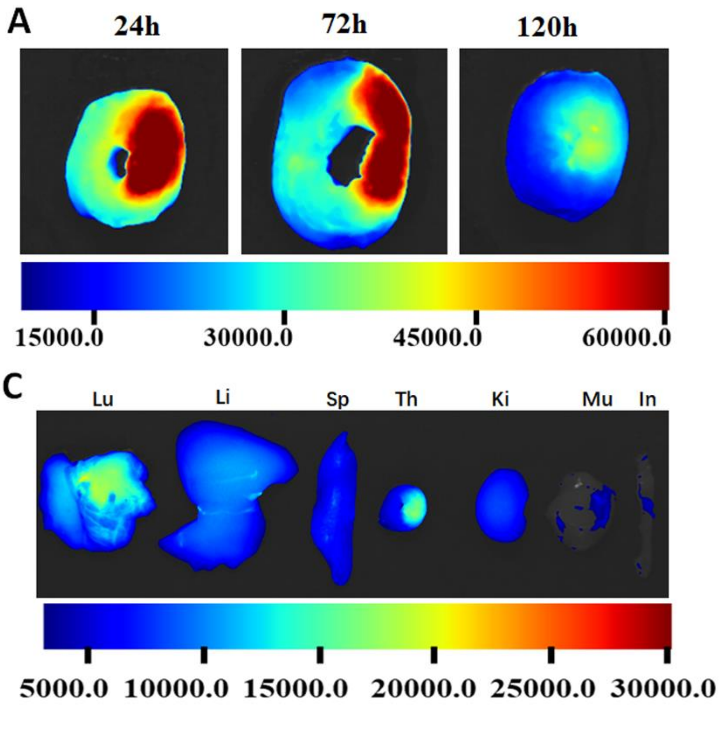

Figure S4. The in vivo imaging stability of IR-780. The representative images A) and quantitative data B) of fluorescence intensity in the transection of the heart at 24h, 72 h,120 h following IR-780 injection (n=3 per group). C)The representative NIR images of the normal organs including lung (Lu), liver (Li), spleen (Sp), kidney (Ki), muscle (Mu) and intestine (In) and the transection of the heart (TH) 2 h post IR-780 injection. D) The fluorescence intensity ratio of the area at risk of the heart and the main organs (n=3 per group).

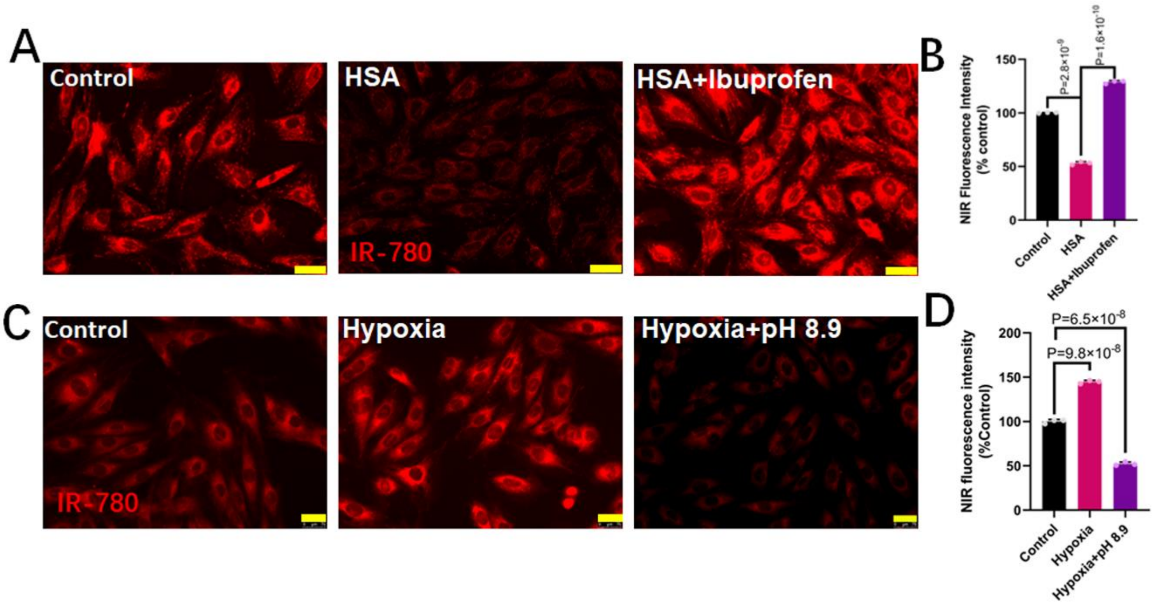

Figure S5. The uptake of IR-780 in HSA and hypoxia conditions. A, B) Representative images and quantitative flow cytometric data of IR-780 uptake in H9C2 cells within HSA or HSA+ ibuprofen solutions. (n=3 per group), Scale bar: 50 µm. C, D) Representative images and quantitative flow cytometric data of IR-780 uptake in H9C2 cells in hypoxia conditions (5% O_2) or hypoxia conditions (5% O_2) and high pH (8.9) (n=3 per group), Scale bar: 75µm. All the *p* values are present in the graphs.

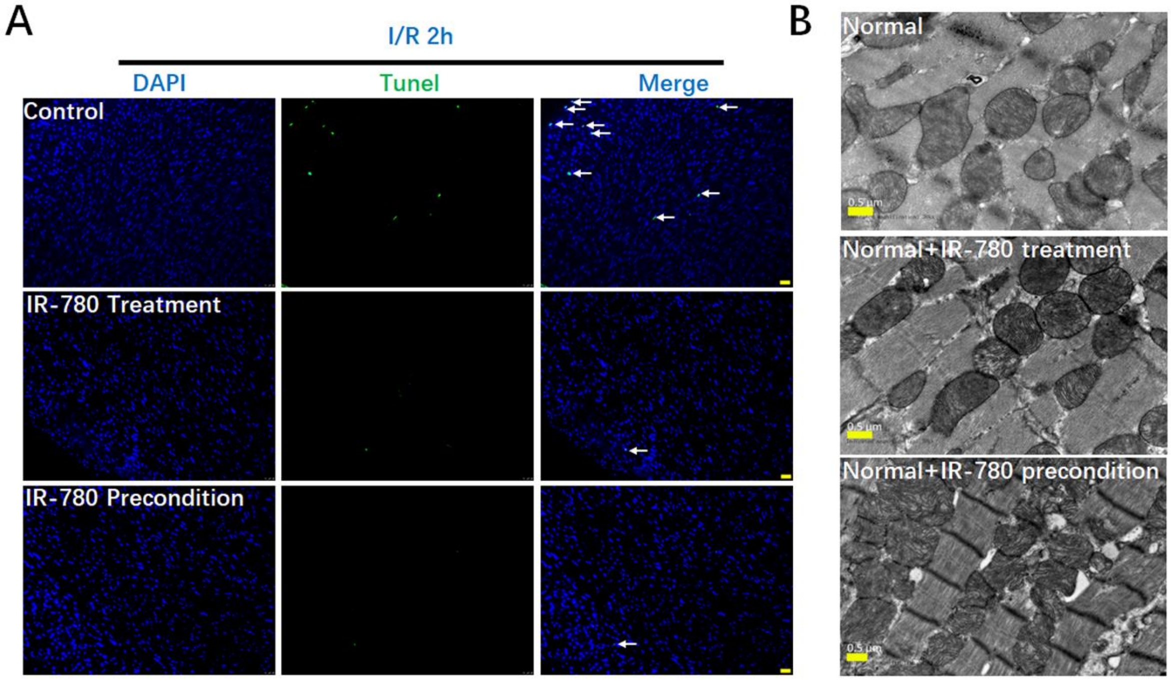

Figure S6. IR-780 decreases the I/R induced apoptosis. A) Representative images related to Figure3 (H). Scale bar: 25μm. Arrow: TUNEL positive cells. B) Representative TEM photographs of the heart tissues exposed to sham, IR-780 treatment and IR-780 precondition. Scale bar: 0.5μm.

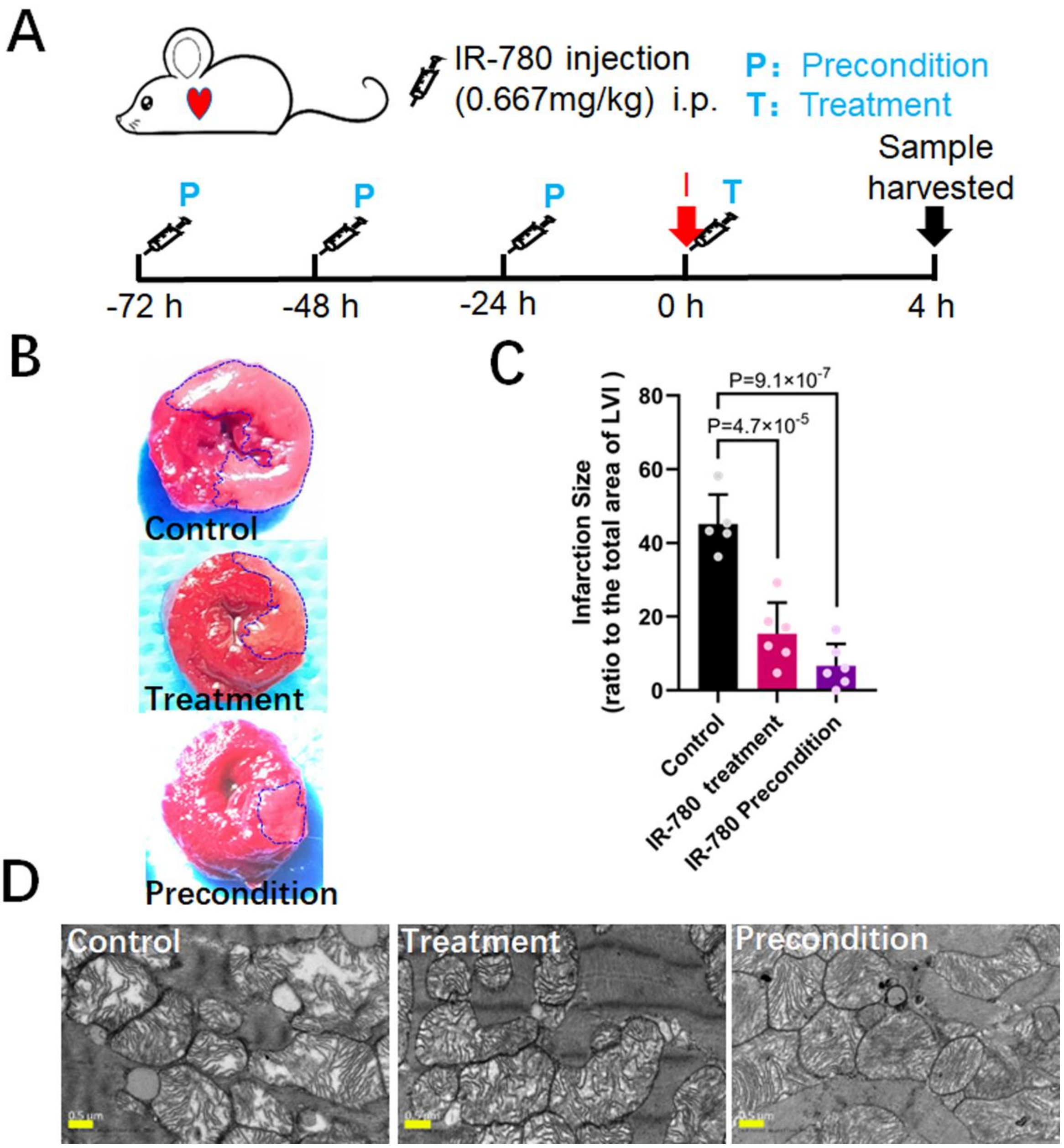

Figure S7. IR-780 protects the heart from ischemia injury. A) Schematic of the IR-780 based cardio-protective protocols in ischemia model. B, C) Representative photographs and quantitative data for infarct size in rats of control (n=5), IR-780 treatment (n=6) and IR-780 precondition (n=6) groups subjected to ischemia. D) Representative TEM photomicrographs of the heart tissues in rats of control, IR-780 treatment and IR-780 precondition groups with ischemia injury. Scale bar, 0.5μm. I: ischemia. All the *p* values are present in the graphs.

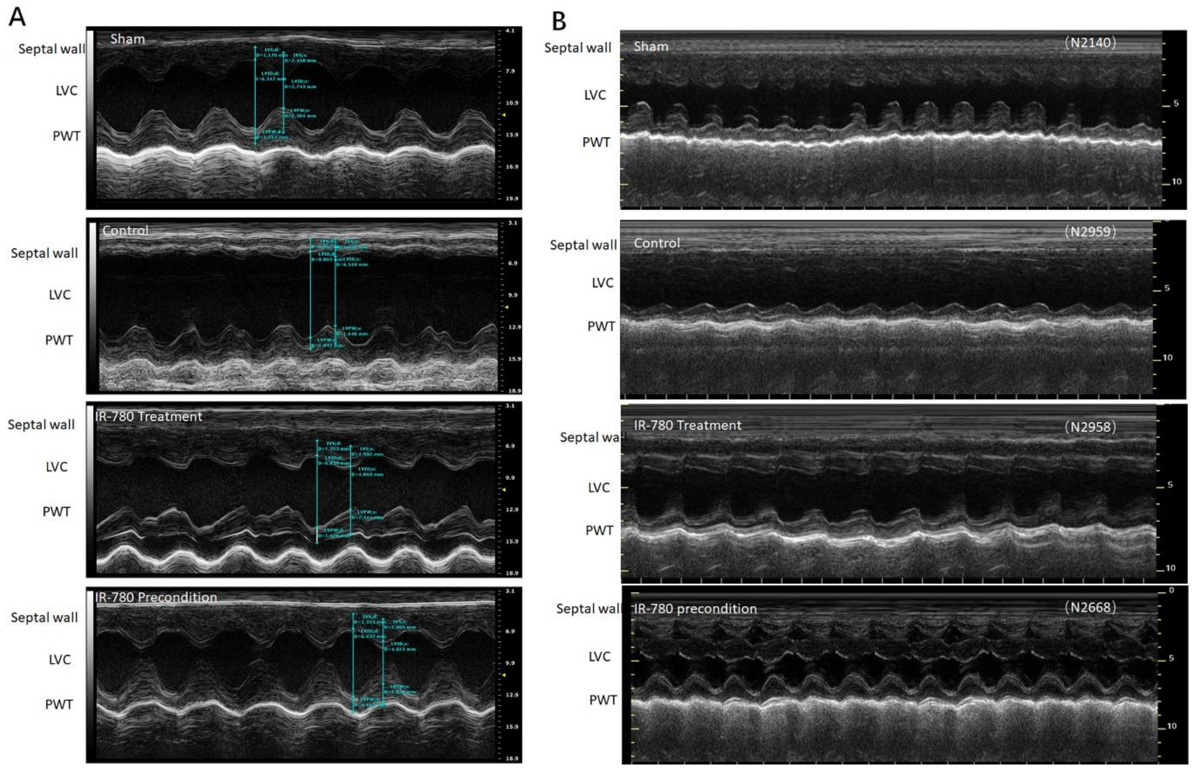

Figure S8. IR-780 improves the cardio-function following I/R injury. Representative images echocardiograms of the hearts 1 months following I/R injury in rat models (A) and pig models (B) related to Figure 3 (L-N) and Figure 3 (Q-S) respectively.

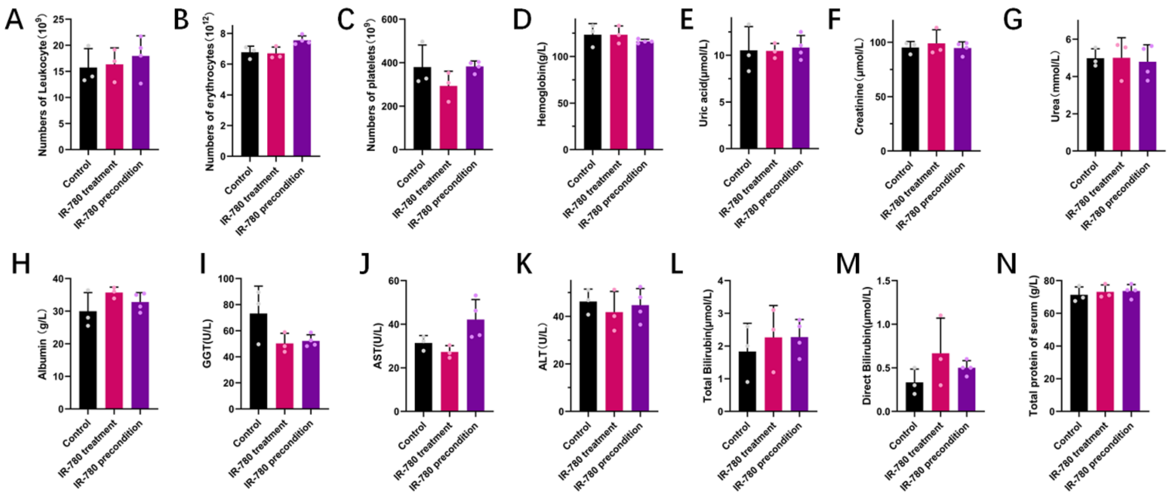

Figure S9. The biosafety of IR-780 to biological organisms in pigs. The quantitative data of the blood routine A-D), renal function E-G), liver function H-N) in control group (n=3), IR-780 treatment group (n=3) and IR-780 precondition group (n=4) 1 month following reperfusion in pig I/R model. GGT, transglutaminase; AST, aspartate transaminase; ALT, Alanine aminotransferase.

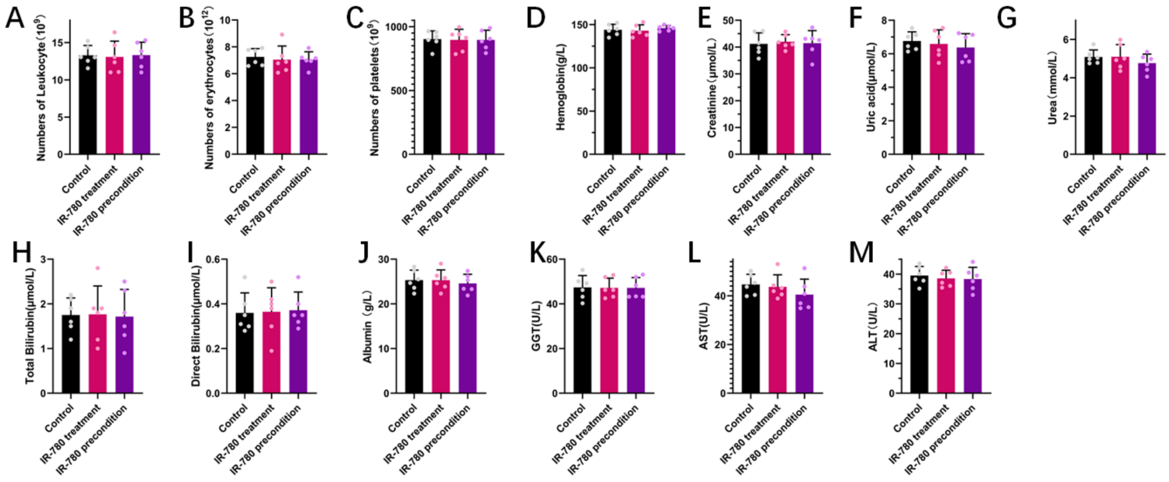

Figure S10. The biosafety of IR-780 to biological organisms in pigs. The quantitative data of the blood routine A-D), renal function E-G), liver function H-N) in control group (n=6), IR-780 treatment group (n=6) and IR-780 precondition group (n=6) 1 month following reperfusion in pig I/R model. GGT, transglutaminase; AST, aspartate transaminase; ALT, Alanine aminotransferase.

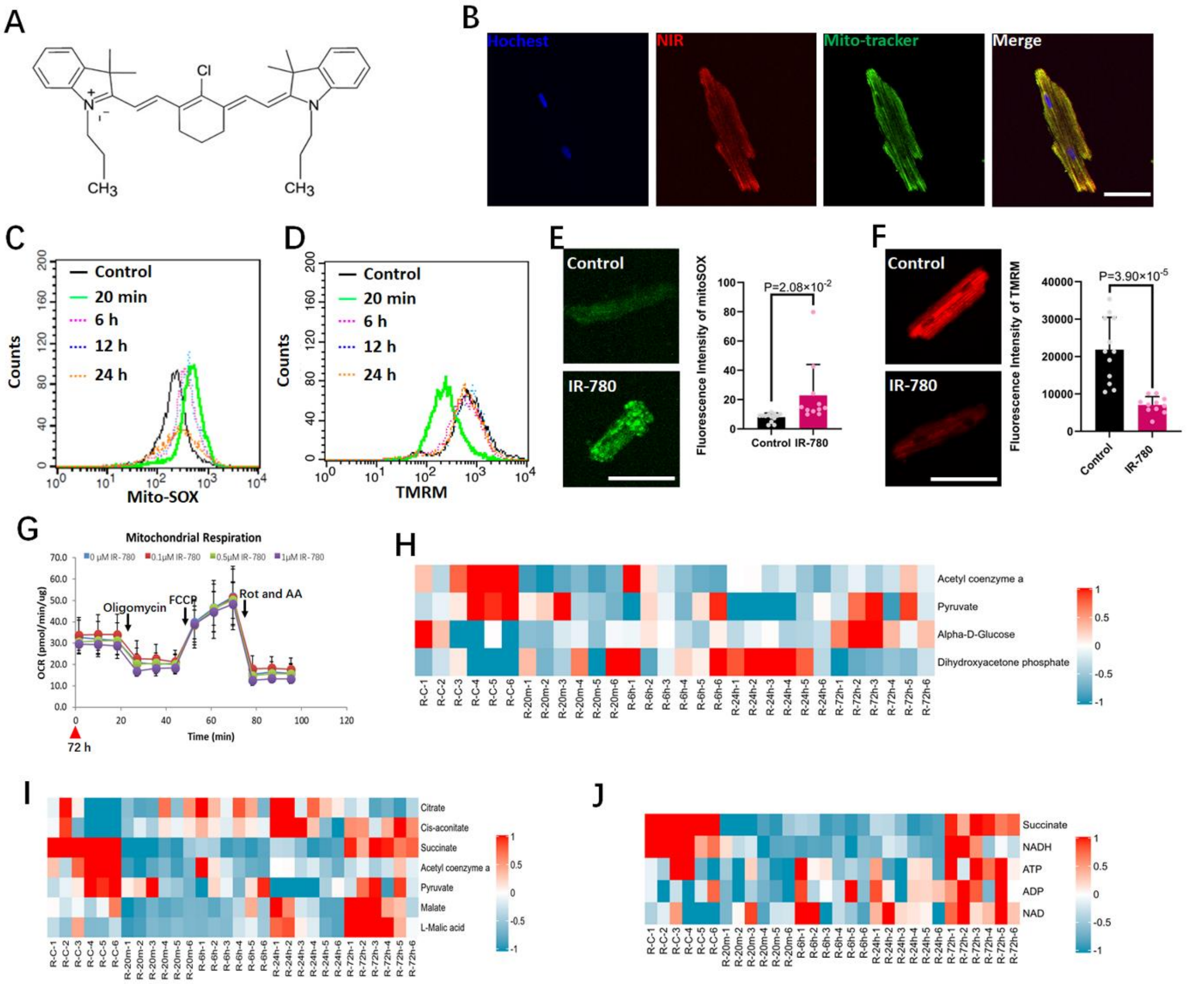

Figure S11. IR-780 depresses the mitochondrial function. A) Chemical structure of IR-780. B) Representative confocal images of the adult rat cardiomyocytes treated with IR-780 (0.1μM) and co-staining with Mito-tracker green. Scale bar, 25μm. Representative images of the mitochondrial membrane potential C) and mitochondrial ROS production D) of the H9C2 cells following IR-780 (0.1μM) administration. E) The representative images and quantitative data of the mitochondrial ROS production and F) the mitochondrial membrane potential in control and IR-780 treated freshly isolated rat cardiomyocytes. Scale bar, 50μm. G) Seahorse analyzer assays showed that H9C2 cells recovered from OCR inhibition 72 h after IR-780 exposure (0, 0.1, 0.5 and 1 μM; represented by four colored lines). Oligomycin (1μM, introduced after 18 min); FCCP (3μM, introduced after 48 min); and a mixture of rotenone and antimycin A (Rot

and AA; 1μM respectively, introduced after 72 min). Heatmap of identified metabolites in Glycolysis H), Citrate cycle (TCA cycle) (I) and Oxidative phosphorylation (J) pathways, n=6 samples per group. All the *p* values are present in the graphs.

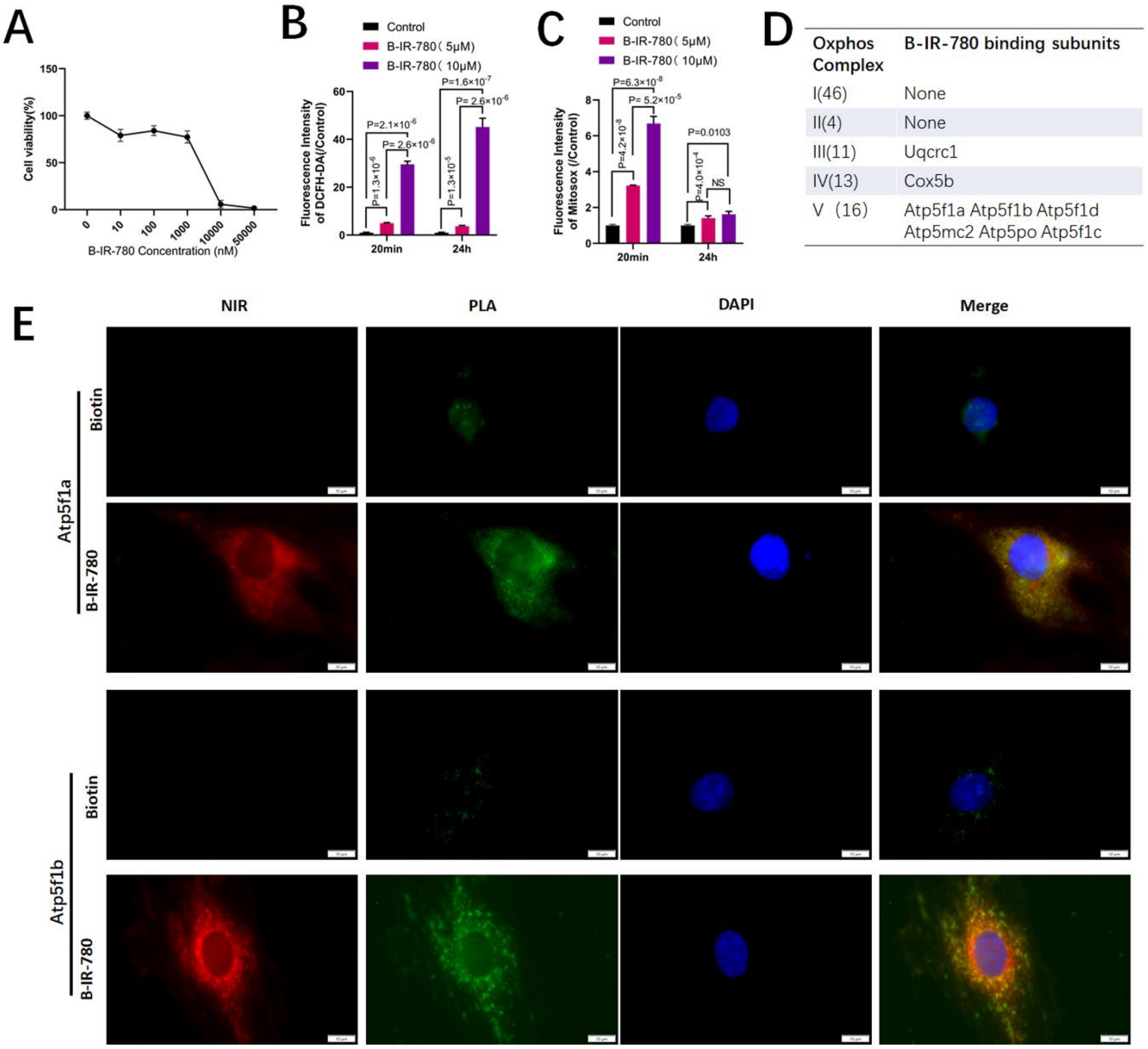

Figure S12. IR-780 regulates the mitochondrial functions by targeting the complex V of the OXPHOS. A) Cell viability assays for H9C2 cells exposed to increasing doses of B-IR-780 over the course of 24 h (n = 6 pre group). B, C) Total cell (B) and mitochondrial (C) ROS production of H9C2 cells at 20 min and 24 h exposed to B-IR-780 (5, 10μM), (n=3 per time point per group). D) B-IR-780 associates with multiple components of the OXPHOS complex. B-IR-780 pull-down of the purified mitochondria from H9C2 cells treated with B-IR-780 followed by mass spectrometry analyses. E) Proximity Ligation Assay for H9C2 cells exposed to biotin or B-IR-780 demonstrates the IR-780-specific interactions with complex V subunit atp5f1a, atp5f1b. Scale bar, 10 μm. All the *p* values are present in the graphs.

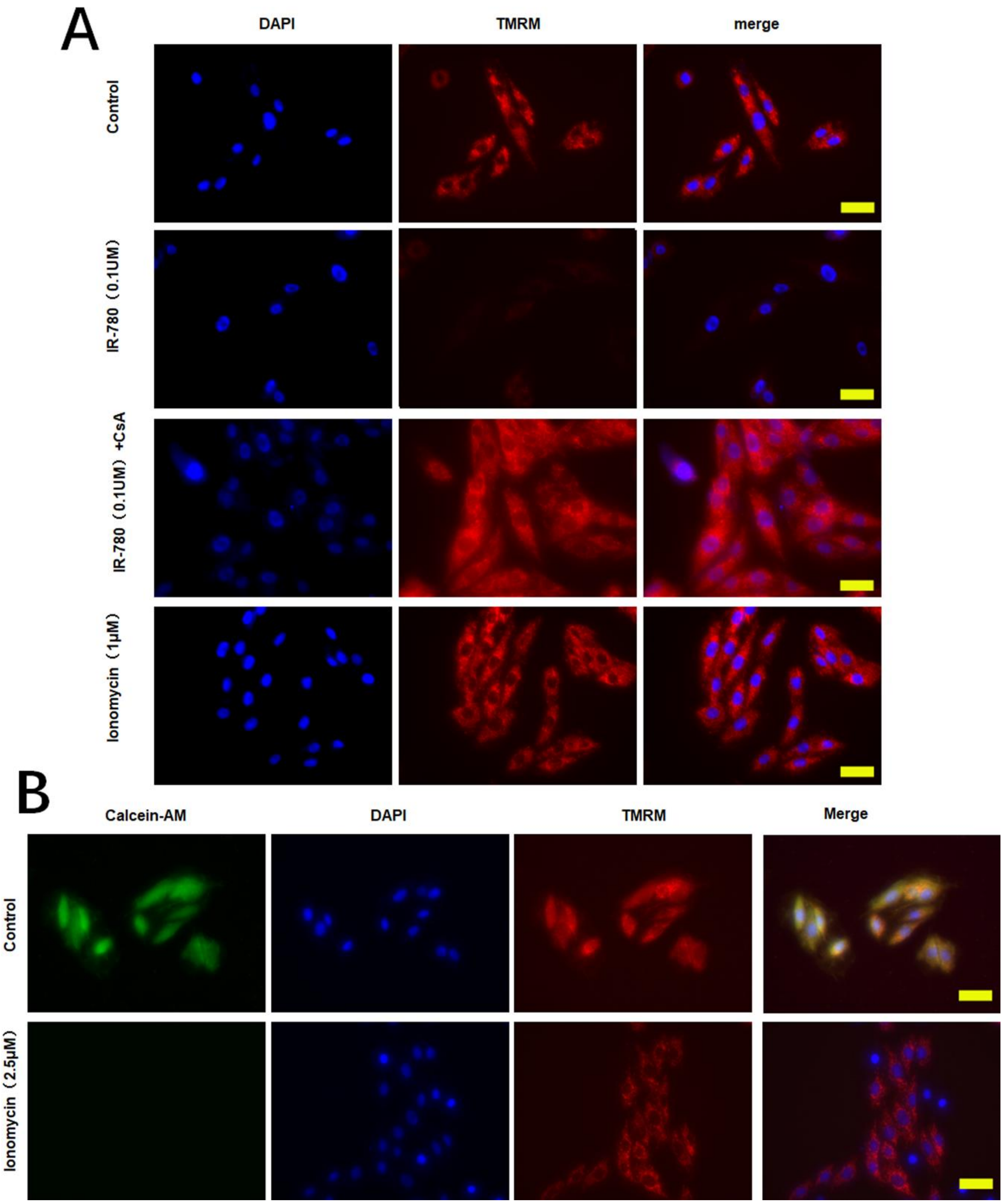

Figure S13. IR-780 decreases the mitochondrial membrane potential. A) Representative images of the mitochondrial membrane potential (TMRM staining) in the H9C2 cells treated with IR-780 (0.1 μM) with or without CsA (10 μM), ionomycin (1 μM). Scale bar, 50μm. B) Representative images of TMRM and Calcein-AM staining in H9C2 cells treated with ionomycin (1 μM). Scale bar, 50μm.

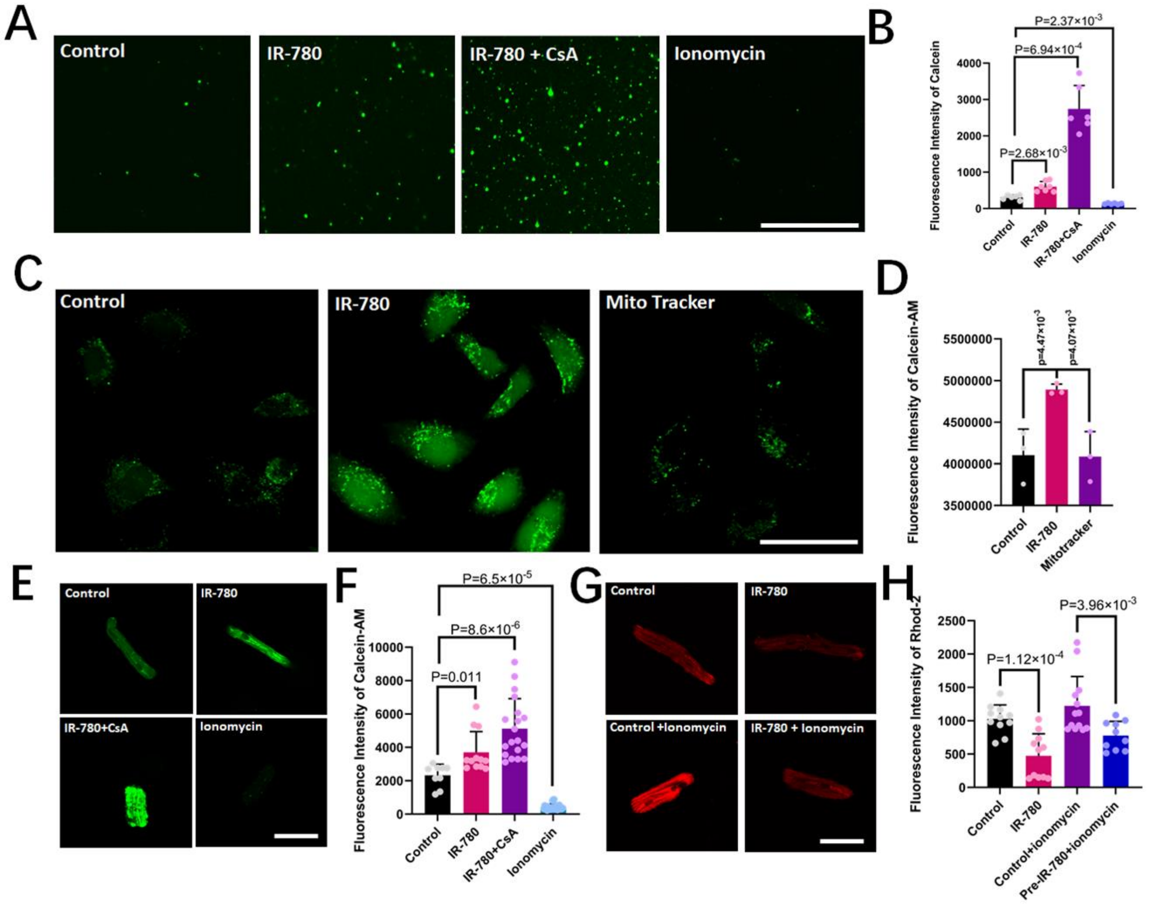

Figure S14. IR-780 inhibit the mPTP activity. The representative images A) and quantitative data B) of calcein fluorescence intensity in control, IR-780, IR-780+ CsA and ionomycin treated freshly isolated mitochondria from adult rat heart tissues. Scale bar, 50µm. The representative images C) and quantitative data D) of the mPTP activity (calcein-AM) in H9C2 cells with PBS, IR-780 and Mito tracker. Scale bar, 50µm.The representative images E) and quantitative data F) of the mPTP activities (detected by calcein-AM) in control, IR-780, IR-780+ CsA and ionomycin treated freshly isolated rat cardiomyocytes. Scale bar, 50µm. The representative images G) and quantitative data H) of the mitochondrial calcium (detected by Rhod-2 AM) in control, IR-780, ionomycin and IR-780+ ionomycin treated freshly isolated rat cardiomyocytes. Scale bar, 50µm. All the *p* values are present in the graphs.

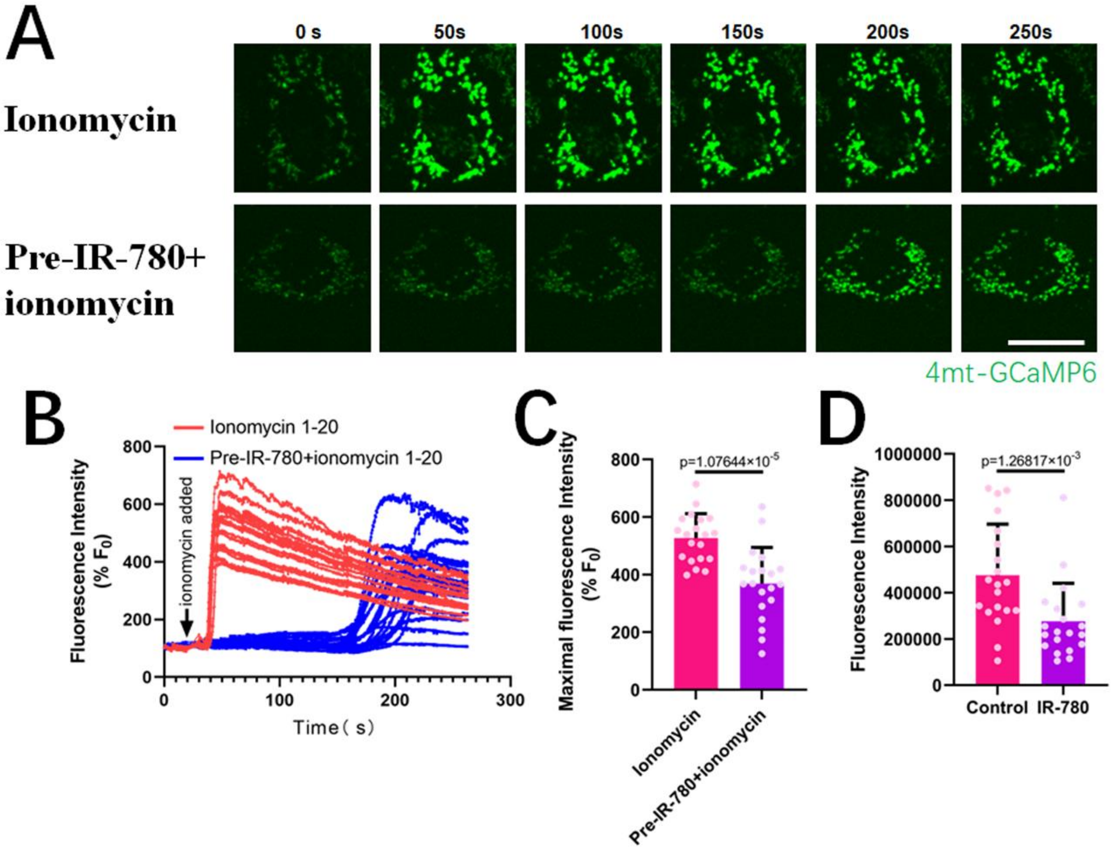

Figure S15. IR-780 decreases the mitochondrial calcium concentrations and inhibits the mitochondrial calcium overload. A) Representative images of the mitochondrial calcium dynamics in a H9C2 cells expressing 4mt-GCaMP6 (green) exposed to ionomycin (1 μM) with or without pre-IR-780 (0.1 μM) treatment (15 minutes before). Scale bar, 50μm. B) The traces of mitochondrial Ca2+ concentration in a H9C2 cells expressing 4mt-GCaMP6 exposed to ionomycin (1 μM) with or without pre-IR-780 (0.1 μM) treatment (15 minutes before). A total of 20 cells in each group were calculated. C) quantitative data of the maximal fluorescence of 4mt-GCamp6 of each cell exposed to ionomycin (1 μM) with or without pre-IR-780 (0.1 μM) treatment (15 minutes before). D) quantitative data of the 4mt-GCamp6 fluorescence in H9C2 cells exposed to IR-780 (0.1 μM). All the *p* vaules are present in the graphs.

Video S1. NIR imaging of the infarcted porcine heart. Myocardial I/R were induced in male farm pigs, and IR-780 (0.667 mg kg^{-1}) were intraperitoneally injected immediately following reperfusion. The NIR signals in the infarct hearts were recorded 48 h following reperfusion.

Reference

[1] B. Liu, A. Li, Y. Qin, X. Tian, M. Gao, W. Jiang, G. Gong, *Journal of Molecular and Cellular Cardiology* **2019**, *136*, 64.

[2] Z. Chen, Z. Wang, T. Jin, G. Shen, Y. Wang, X. Tan, Y. Gan, F. Yang, Y. Liu, C. Huang, Y. Zhang, X. Fu, C. Shi, *Theranostics* **2019**, 9, 6797.

[3] X. T. Zheng, C. M. Li, *Biosensors & bioelectronics* **2010**, 25, 1548.
